# Contributions of Ecosystem Services to Human Well-Being in Puerto Rico

**DOI:** 10.3390/su12229625

**Published:** 2020-11-18

**Authors:** Susan Harrell Yee

**Affiliations:** Gulf Ecosystem Measurement and Modeling Division, US Environmental Protection Agency, 32561 Gulf Breeze, FL, USA

**Keywords:** human well-being, ecosystem services, economic services, social services, spatial interpolation

## Abstract

Ecosystem services, including availability of greenspace, clean air, and clean water, can have benefits to human well-being, but their relative importance compared to economic or social services is often overlooked. In Puerto Rico, for example, improving community well-being, including economic and cultural opportunities, human health, and safety, are often overarching goals of environmental management decisions, but the degree to which improvements in ecological condition and provision of ecosystem services could impact local communities is complicated by wide variation in social and economic conditions. This study quantifies and maps neighborhood-scale indicators of human well-being and ecosystem services for Puerto Rico to better understand the degree to which ecosystem services provisioning, alongside co-occurring social and economic services, explains variability in a number of indicators of human well-being. In Puerto Rico, variability in indicators of human well-being were predominately explained by economic services related to accumulating income and personal savings, and social services, including availability of family services, healthcare services, and access to communication technology. Despite the large explanatory power of economic and social services, however, the analysis detected that substantial portions of well-being, in particular education and human health, could be explained by variability in ecosystem services over space and time, especially availability of greenspace. Linking ecosystem services to multivariate elements of human well-being can serve to complement more traditional community planning or environmental management efforts by helping identify potential unintended consequences or overlooked benefits of decisions.

## 1. Introduction

Human well-being is often implied or directly identified as an overarching goal of natural resources management [1,2], environmental risk assessment [3], and community planning [4]. Estuarine management plans, for example, are increasingly framing ecosystem restoration and other decisions within the context of benefits to the well-being of stakeholders by maintaining or improving provisioning of ecosystem services [5,6]. Ecosystem goods and services can have overlooked benefits that are important to community well-being, including physical and mental health [7,8], wealth [9], education [10], culture and spirituality [11], and social connections [12]. Although several efforts have been undertaken to connect the provisioning of ecosystem services to measures of human well-being [13–15], the relative importance of ecosystem services compared to economic or social services is less clear [16].

In the United States island territory of Puerto Rico, for example, a large proportion of island residents live within the watershed of the San Juan Bay estuary, one of the most heavily urbanized estuary watersheds in the United States [17]. In consideration of this, management objectives of the San Juan Bay Estuary Program include improving multiple aspects of community well-being for people living in the watershed, including economic opportunities, cultural heritage, human health, education, public safety, social engagement, and good governance, in addition to more typical ecological goals of improving water quality and habitat [17]. Even in the densely populated Puerto Rican capital city of San Juan, vegetation covers about 42% of the land, and neighborhoods derive numerous benefits from green infrastructure including food items cultivated in their yards and pollution removal upstream [18], factors which could be considered when developing sustainable development and resource conservation strategies [19]. More recently, in the aftermath of hurricane Maria in 2017, community and conservation leaders in Puerto Rico are increasingly considering how green infrastructure solutions can reduce flood risk, improve livability, and support resilience of the island to future risks [20]. Predicting the potential benefits of environmental management or green infrastructure decisions on human well-being, however, is complicated by the widely varying socio-economic conditions throughout the island [18,21].

**Figure 1.**
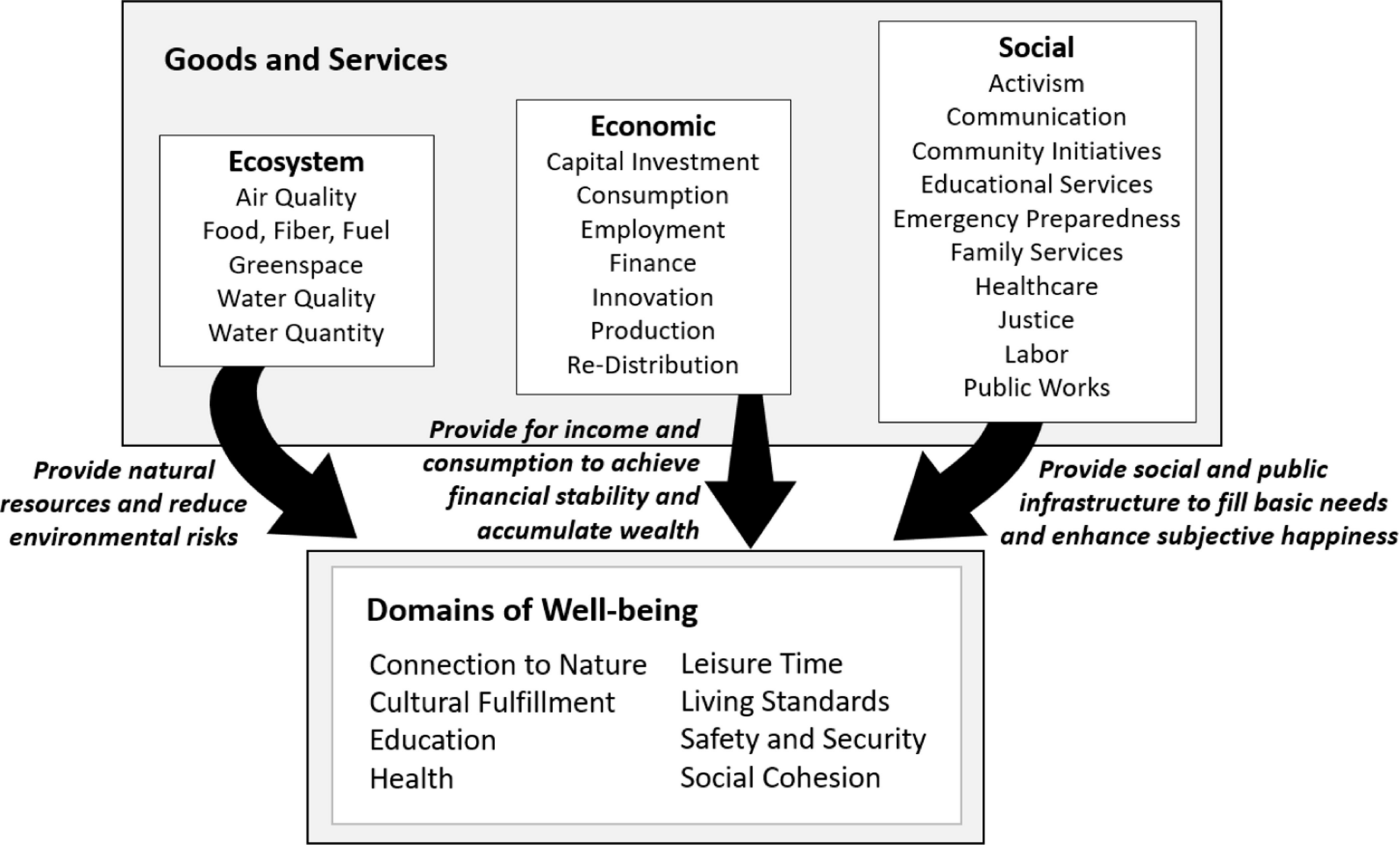
Conceptual diagram of the indicator categories in the Human Well-Being Index (HWBI) framework illustrating how changes in ecosystem, economic, and social goods and services influence elements of human well-being (modified from [24]).

Integrated assessment approaches that link economic, social, and ecological decision alternatives to human well-being may be valuable in estimating potential benefits and trade-offs in terms that are meaningful to people living in a community [16]. Well-being is multifaceted [22], and composite indices provide a comprehensive approach to defining and measuring well-being in terms of multi-dimensional components [23–29]. The United States Human Well-Being Index (HWBI), for example, is a hierarchical index of well-being, composed of sub-indices representing eight domains: connection to nature, cultural fulfillment, education, health, leisure time, living standards, safety and security, and social cohesion [23]. Changes in HWBI are assumed to be driven by ecosystem, economic, and social services that deliver goods, support, and assistance to society (Figure 1; [16,30]). At a national scale for the fifty states of the United States, the HWBI conceptual framework identifies metrics and indicators of ecosystem services (e.g., clean air, water quality and quantity), economic services (e.g., infrastructure investment, job creation), and social services (i.e., healthcare, emergency preparedness) that drive changes in well-being [24]. A series of statistical relationships have previously been derived for the U.S. fifty states to quantitatively describe how changes in economic, social, and ecosystem services relate to changes in the eight HWBI domains of well-being [16,30]. These statistical models provide a basis for understanding how increases or decreases in the availability or quality of economic, social, or ecosystem services may impact multi-dimensional components of well-being.

In this study, we apply the U.S. HWBI framework [16] to model the relationships between economic, social, and ecosystem services and human well-being for Puerto Rico. Puerto Rico is a territorial commonwealth of the United States; residents have U.S. citizenship and the economy operates within the U.S. financial system [31]. Puerto Rico has a land area of 9104 km^2^, approximately the size of the third smallest U.S. state of Connecticut. Yet Puerto Rico is densely populated, with more than two-thirds of residents living in the metropolitan area of San Juan, the 21st most populated urban area in the United States according to the 2010 US Census [32]. The population of Puerto Rico has declined from approximately 3.81 million in 2000 to 3.47 million in 2017 [32]. The transition of the economy in the mid-20th century from agriculture to industry and loss of job opportunities for rural residents, exacerbated by the post-2005 economic recession, has contributed to a mass migration of island residents to the mainland U.S. that has led to net population loss in recent years ranking among the highest rates of loss globally [33]. The Puerto Rican economy is considerably less affluent than the mainland U.S. [31], with high public debt and median incomes 62% lower than the mainland [32]. A composite human well-being index previously developed for Puerto Rico based on the HWBI methodology [34] ranked in the bottom ten when compared to the fifty U.S. states for 2000–2010, but domain scores for connection to nature and cultural fulfillment were much higher for the island. Although part of the U.S., it is unclear whether the statistical relationships between services and well-being derived for the U.S. fifty states [16] are broadly applicable to Puerto Rico, particularly given major differences in land mass, population density, and socio-economic disparities between the island and the mainland U.S. Further analysis is needed to determine the degree to which factors, such as water quality, healthcare, or employment opportunities, which correlated with components of well-being for the U.S. fifty states [16], similarly explain temporal and spatial variability in well-being for Puerto Rico.

This study quantifies and maps neighborhood-scale indicators of human well-being and ecosystem services for Puerto Rico to better understand the degree to which ecosystem services provisioning explains variability in elements of human well-being. The U.S. HWBI framework is used to quantify a suite of economic, ecosystem, and social services indicators (Figure 1; [23,24]) and evaluate their ability to explain variability in previously developed indicators of well-being for Puerto Rico [34]. The HWBI domains have been shown through stakeholder engagement workshops to resonate broadly with U.S. communities [4], and align well with environmental management objectives in Puerto Rico, including human health, public safety, economic and cultural opportunities (e.g., San Juan Bay estuary management plan; [17]). Spatial interpolation methodologies are applied to fill in missing data and reconcile all data to similar spatial scales [35,36]. Multiple regression and model averaging are used to assess the relationships between economic, ecosystem, and social services and the elements of human well-being. To better understand robustness of results and explain underlying patterns, statistical analyses were evaluated not only aggregated indicators, but also for individual metrics contributing to each indicator. By evaluating the amount of variability explained by each of the three types of services in regressions, this study estimates the relative contributions of ecosystem services, in particular, to spatial and temporal differences in human well-being.

## 2. Materials and Methods

### 2.1. Human Well-Being Domains and Services Scores

The U.S. fifty state HWBI [23] was recently adapted to Puerto Rico municipios (county-equivalent; [34]) and census tracts [36] by substituting Puerto Rican data to calculate indicators for each well-being domain. Here, the same process is followed to calculate indictors for economic, ecosystem, and social services for Puerto Rico. Briefly, yearly data from 2000–2017 for metrics representing each indicator were obtained from sources at the best available spatial scale (e.g., Puerto Rico, region, municipio, or census tract). Puerto Rico is comprised of 909 census tracts within 78 municipios, with a mean tract area of 10.9 km^2^ and mean tract population size of 3929 people. Metric data representing each indicator were obtained from publicly available web-based databases where possible, with some data extracted from peer-reviewed literature, reports, or other publications. Where possible, the original U.S. HWBI domains [23] or services [24] metrics and data sources were used, or a comparable source for the same metric. If the original metric was not attainable for Puerto Rico, surrogate metrics were identified that maintained the intention of the indicator [34]. If multiple sources were available for a metric, each capturing distinct but relevant information (e.g., one has better temporal coverage, the other better spatial coverage), both were included as separate metrics so that the average of the two data sources would be incorporated into the indicator calculation. If a suitable surrogate or data source could not be identified, a metric from the original U.S. HWBI was dropped, but maintaining at least one metric per indicator. In total, this study identified 75 metrics representing the well-being domain indicators (Table 1), 37 metrics representing economic service indicators (Table 2), 25 metrics representing ecosystem services indicators (Table 3), and 101 representing social services indicators (Table 4).

Missing years were temporarily imputed for the Puerto Rico data set using carry-forward substitution [34]. Only 8 of 75 domain metrics had a complete yearly set of data for the entire study period 2000–2017, with an additional 28 domain metrics having data for at least half of the years. For services, 16 out of 37 economic services metrics, 8 out of 25 ecosystem services metrics, and 72 out of 101 social services metrics had yearly data for at least half of the years in the study period. Missing spatial data were spatially imputed from available coarser-scale data at the same location (e.g., replacing missing municipio-scale data with regional data). Only 9 out of 75 domain metrics and 44 out of 163 services metrics were available at the census tract scale, with most other metrics available at the municipio scale. Therefore, in order to form a complete and uniformly scaled data set for each census tract from 2000–2017, census tract-scale data were interpolated from coarser-scale data using a downscaling methodology derived from MERLIN (Model for External Reliance of Localities IN coastal management zones) [35,36]).

**Figure 2.**
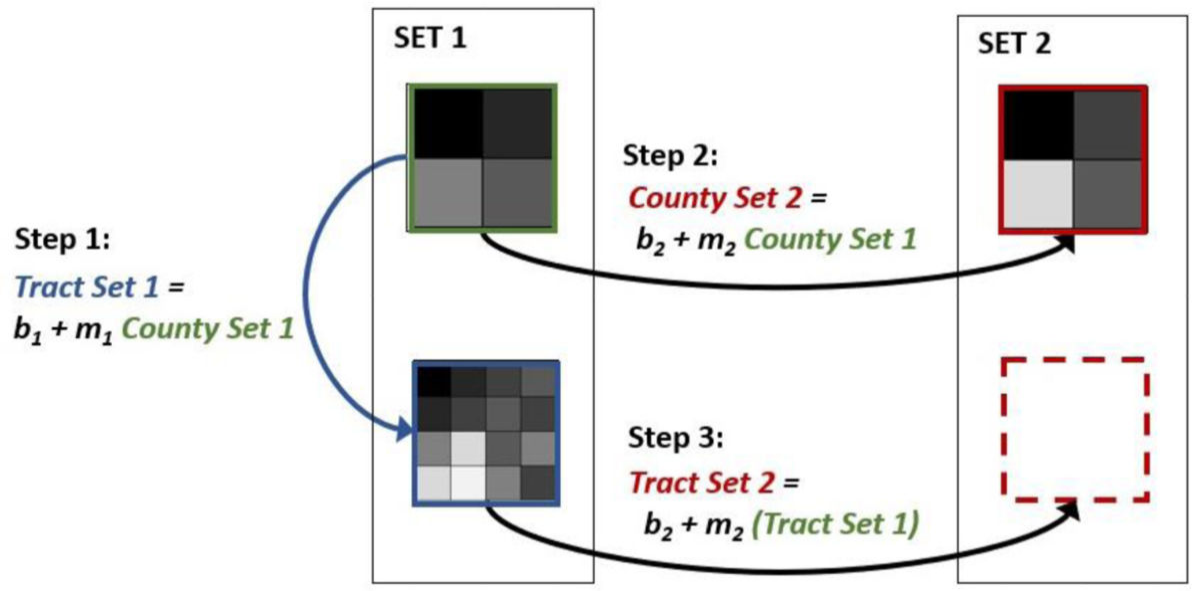
Simplified illustration of the steps in the modified Model for External Reliance of Localities IN coastal management zones (MERLIN) spatial interpolation [36], which uses (Step 1) the relationship between county-scale data and tract-scale data to generate corrected tract-scale data as input into (Step 2) relationships between county-scale metrics to (Step 3) predict missing tract-scale data. Set 1 metrics are available at both geographic scales; Set 2 metrics are available only at the coarser scale.

A modified version of the MERLIN approach (Figure 2) was previously used to downscale the municipio-scale well-being domain metrics for Puerto Rico [34] to the census tract scale from 2000–2017 [36]. Here, the same modified MERLIN methodology [36] used for domains is implemented to downscale the services metrics. Briefly, relationships between Set 1 metrics available at both the tract scale and county scale (Step 1, Figure 2), are used to generate input into relationships between Set 1 and Set 2 county-scale metrics (Step 2, Figure 2) to predict missing tract-scale data for Set 2 metrics from their known county-scale values (Step 3, Figure 2). The Step 1 and Step 2 relationships were calculated using forward step-wise general linear mixed models, with year as a random grouping effect to account for potential differences in relationships between years, and comparing models by using Akaike Information Criteria, or AIC, with the best model having the smallest AIC [126]. Because the predicted downscaled data are based on metrics where more complete yearly data may be available, the approach has the additional effect of filling in, or smoothing, missing temporal data which had been temporarily imputed as a carry-forward step-function prior to conducting regressions. Regressions were done in R (www.r-project.org) using the library ‘nlme’, and using ‘r.squaredGLMM’ of the library “MuMIn” to evaluate model goodness of fit. Accuracy of the downscaling approach was evaluated in two ways [36]. First, for metrics with known tract-scale data, the approach should be able to accurately recreate the tract-scale data when the tract-scale data are used to predict itself. Second, the approach should be able to accurately maintain county-scale averages, in that predicted tract-scale metrics averaged to the county-scale should match county-scale data. Predicted versus actual values were compared by calculating Pearson’s correlation coefficients (*r*) for each metric.

Following the original HWBI methodology [23,24,34], downscaled (tract-scale) data for each year were then used to calculate yearly aggregate indicator, service, and domain scores for each tract from 2000–2017. Metrics are normalized to reduce the influence of extreme outliers and standardized between 0.1 and 0.9. Metrics are reversed as needed to maintain a positive relationship between each metric and either well-being or services provisioning (e.g., mortality rates would be inversely associated with positive well-being). Indicator scores are then calculated as the arithmetic mean of the standardized metrics for each indicator, and domain or service scores as the arithmetic mean of indicators within each domain or service. An overall composite HWBI score and overall composite Economic, Ecosystem, or Social Service scores were calculated as the arithmetic mean of all domain or service scores in each census tract or year. To help visualize trends over time, the slope of domain or service scores over time from 2000 to 2017 in each tract was estimated using simple linear regression, where a positive slope indicates a general increase over the time period and a negative slope indicates a general decline.

### 2.2. Statistical Relationships between Services and Domains

To evaluate the relationships between service scores and domain scores, forward step-wise multiple regressions were used to identify the set of models that best explained variability in observed scores. Because tract-scale scores within a municipio or between years are not independent, this study implemented multiple regressions as general linear mixed models, with year and county as random grouping effects. Candidate models were fit using maximum likelihood estimation (MLE; [127,128]). This study used the corrected AIC (AIC_c_) to compare candidate models, with the best model having the smallest AICc [126]. At each step, variables were added to each model if they improved the variance explained by the fixed effects [129]. Additionally, variance inflation factors (VIF) were used to check for collinearity, rejecting the addition of variables at each step that caused VIF > 3 [130]. All models that met these two criteria, and were within ΔAIC < 4 of the model with the smallest AIC, were retained at each step. This study used model averaging to estimate the mean parameter value for each variable included in the final set of top models [131]. Model weights for averaging were based on the AICc weights [126]. Calculations were done in R (www.r-project.org) using ‘lmer’ of the library ‘lme4′ to fit models, and ‘dredge’, ‘model.avg’, ‘r.squaredGLMM’ of the library “MuMIn” for model comparison, model averaging, and variance explained. Final models were calculated using restricted maximum likelihood (REML) estimation, which is unbiased compared to MLE because it removes degrees of freedom and underestimation of variance components associated with including fixed effects, but can therefore not be used for comparing fixed effects by AIC [127,128]. For large sample sizes, as in the analysis here, the MLE bias is small and there was minimal difference between final parameter estimates given by the two approaches.

To estimate the relative importance of economic, ecosystem, or social services, Shapley Scores were calculated [132]. The Shapley score of a predictor variable describes the difference in variance explained by adding that variable to a model containing a subset of all other predictor variables. The score is calculated by averaging the differences over all possible combinations of models with and without that predictor (e.g., the single predictor variable vs. the null model; the full model with the variable of interest vs. the set of all other predictor variables). The sum of Shapley scores across all predictor variables is then roughly equivalent to the total marginal R^2^ of the full model (i.e., the variance explained by all the fixed effects; [129]). Therefore, we estimated the relative importance of economic, ecosystem, or social services variables by summing the Shapley scores of predictor variables within each service type relative to the sum total of all Shapley scores. Shapley scores and relative importances were calculated for each model in the set of top models and then averaged using the AICc weights across all top models.

Multiple regressions were run and estimated relative importance was calculated for each of the eight domains of well-being, with the 7 economic, 10 social, and 5 ecosystem services as potential predictor variables (Figure 1), as measured by the aggregated indicators defining each domain (Table 1) or service (Tables 2–4). Because indicators within a domain represent different elements of well-being, are not necessarily positively correlated, and therefore do not necessarily respond similarly to services, multiple regressions were additionally run for 22 of the 25 domain indicators to assess in finer detail which elements of well-being were explained by changes in services. Three domain indicators (time spent on leisure, perceived safety, and risk to safety) were not analyzed due to limited data availability. Finally, this study explored which individual metrics might be explaining relationships between services and domains. However, because the large number of metrics (75 for domains; 163 for services) made running metric-level regressions impractical, Principal Components Analysis (PCA) with a varimax rotation was first used to combine strongly correlated metrics into composite PC scores for each domain or service. PCA were based on centering and scaling downscaled metric scores. Kaiser criteria >1, a cutoff of at least 50% cumulative variance explained, along with an examination of scree plots, was used to determine the number of PCs to include. Multiple regressions were then run on these domain-level PC scores with service-level PC scores as predictor variables. PCAs were calculated in R using ‘principal’ of the library ‘psych’.

## 3. Results

### 3.1. Downscaling

The modified MERLIN downscaling methodology was able to accurately recapture known tract-scale values and county-scale averages (Table 5). For metrics available at the tract scale, Step 1 regressions between tract-scale data and county-scale data were often one-to-one, and always strongly significant (*p* < 0.01, R^2^ > 0.35). Fit less than R^2^ = 1 generally indicated that tract-scale and county-scale data had somewhat different temporal or spatial coverages, and the Step 1 regression was needed to create a corrected set of tract-scale data for use in Step 2. This was particularly common with data obtained from the U.S. Census, where tract-scale data were generally available only in later years of the time period, while county-scale data were available in earlier years. For metrics not available at the tract scale, most metrics could be significantly predicted in Step 2 by at least one tract-available metric (*p* < 0.01, mean R^2^ > 0.56). Metrics under connection to nature and cultural fulfillment had the lowest predictive accuracy (0.30 ≤ R^2^ ≤ 0.41), predominately due to the lack of spatial and temporal data available for these metrics (Table 1). The predicted tract-scale values from the downscaling were strongly correlated with known tract-scale values (0.83 ≤ *r* ≤ 1.0), validating the downscaling methodology. Furthermore, estimated county averages from predicted downscaled data were strongly correlated with known county-scale values (0.72 ≤ *r* ≤ 1.0). Given the overall accuracy of the downscaling methodology, this study proceeded with all further analyses using the predicted downscaled (i.e., tract-scale) data for all years.

### 3.2. Spatial and Temporal Patterns of Well-Being and Services

**Figure 3.**
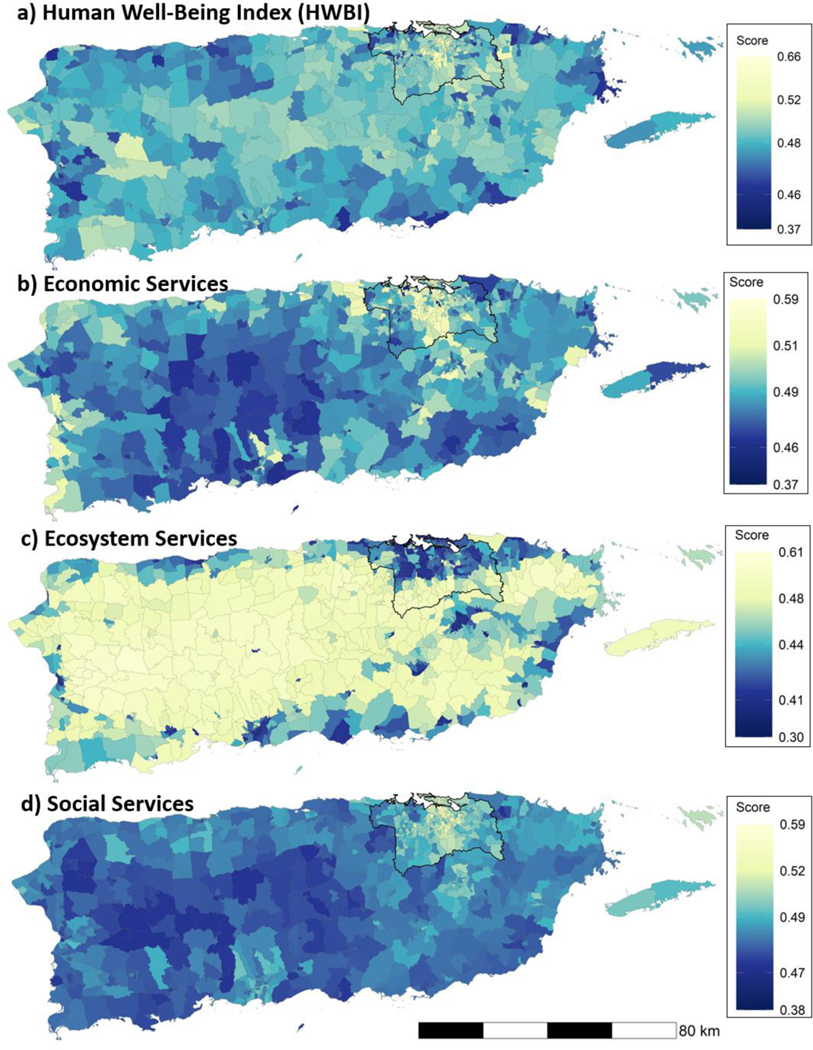
Mean annual (2000–2017) scores in each census tract for (**a**) HWBI as the average of eight well-being domain scores, and (**b**) economic, (**c**) ecosystem, and (**d**) social services as the average of service scores. Enumeration in each legend indicates the quartiles across all years and census tracts for HWBI or each service (dark blue, < 25th percentile; blue, 25–50th percentile; light green, 50–75th percentile; yellow, 75–100th percentile). The San Juan metropolitan area, comprising six municipios, is outlined in black.

On average, the eight domains of well-being were highest in the metropolitan area in northeastern Puerto Rico around the capital of San Juan (Figure 3a), where values of economic services (Figure 3b) and social services (Figure 3d) were also generally highest. In contrast, ecosystem services (Figure 3c) tended to be highest away from the northeastern metropolitan area in the central, less developed, and more forested parts of the island. Maps for individual well-being indicators and services are available as Supplementary information.

**Figure 4.**
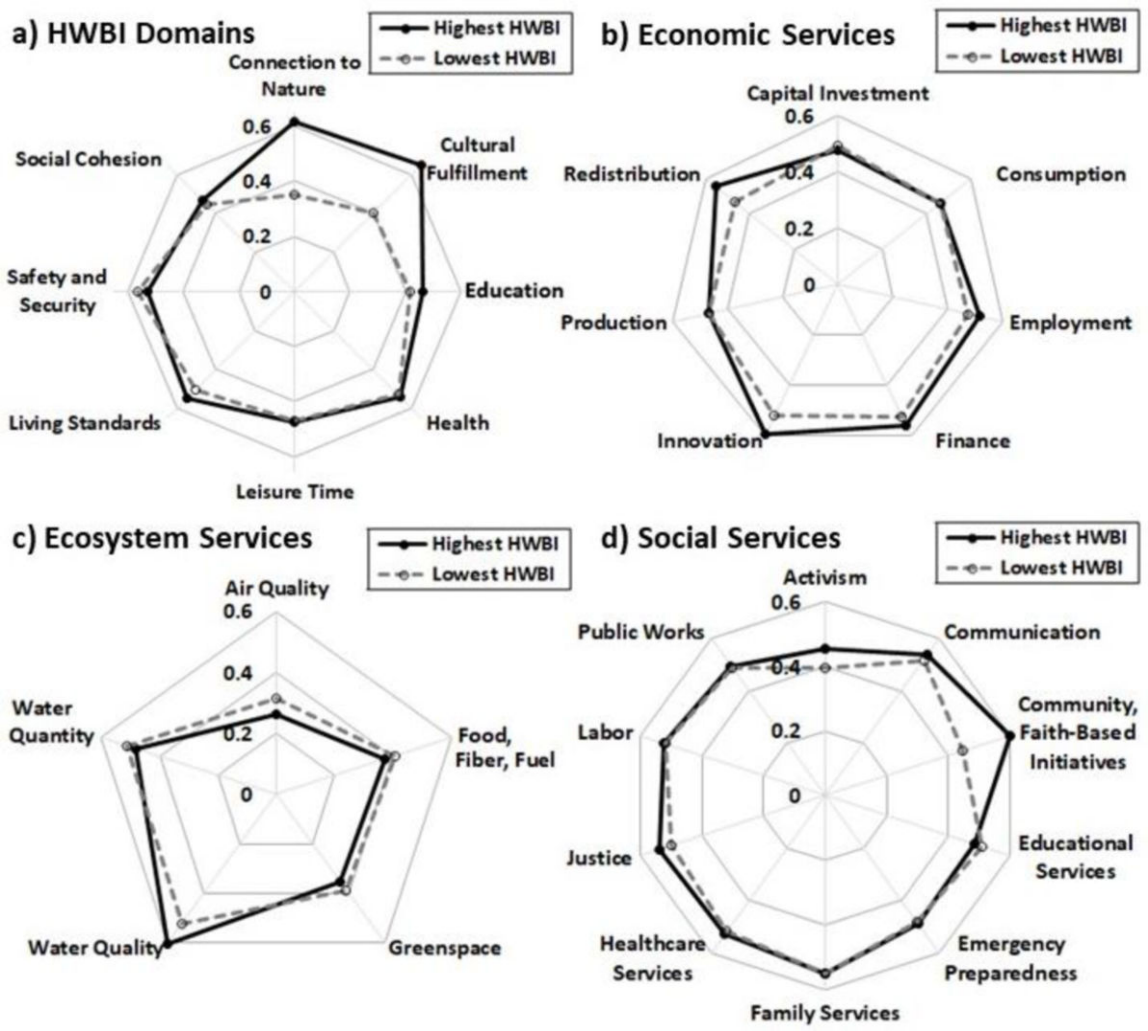
Mean scores across all years 2000–2017 for each HWBI domain (**a**) and each service (**b**–**d**), among census tracts classified as having the lowest (<25th percentile; dashed lines) or highest (>75th percentile; solid lines) mean annual HWBI scores mapped in Figure 3a.

Census tracts with the highest mean HWBI scores were generally higher in connection to nature and cultural fulfillment, and somewhat higher in education and living standards than census tracts with the lowest mean HWBI scores (Figure 4a). Composite domain scores for health, leisure time, and social cohesion were fairly consistent island-wide. Census tracts with high mean HWBI scores tended to be higher in most indicators of health, especially personal well-being (highest HWBI tracts: mean score 0.54; lowest HWBI tracts: mean score = 0.49) and physical/mental health (highest HWBI tracts: mean score 0.54; lowest HWBI tracts: mean score = 0.48), but were generally lower in perceptions of care (highest HWBI tracts: mean score 0.51; lowest HWBI tracts: mean score = 0.50). Indicators for social cohesion were mixed among census tracts with highest compared to lowest mean HWBI scores, with attitude toward community (highest HWBI tracts: mean score 0.46; lowest HWBI tracts: mean score = 0.42), social engagement (highest HWBI tracts: mean score 0.58; lowest HWBI tracts: mean score = 0.51), and social support (highest HWBI tracts: mean score 0.55; lowest HWBI tracts: mean score = 0.51) trending higher, but democratic engagement tending to be lower (highest HWBI tracts: mean score 0.46; lowest HWBI tracts: mean score = 0.50). Composite safety and security scores were generally lower in census tracts with highest mean HWBI scores (Figure 4a).

Census tracts with the highest mean HWBI scores tended to have higher levels of economic services, particularly employment, finance, innovation, and redistribution of wealth (Figure 4b). Social services also typically were higher in census tracts with high mean HWBI scores, particularly in terms of activism, community initiatives, and justice, although many social services were fairly consistent across the island (Figure 4d). In contrast, census tracts with the highest provisioning of ecosystem services generally had the lowest mean HWBI scores (Figure 4c). Only water quality ecosystem services were higher in census tracts with high mean HWBI scores, while air quality, food/fiber provisioning, greenspace, and water quantity all tended to be lower.

**Figure 5.**
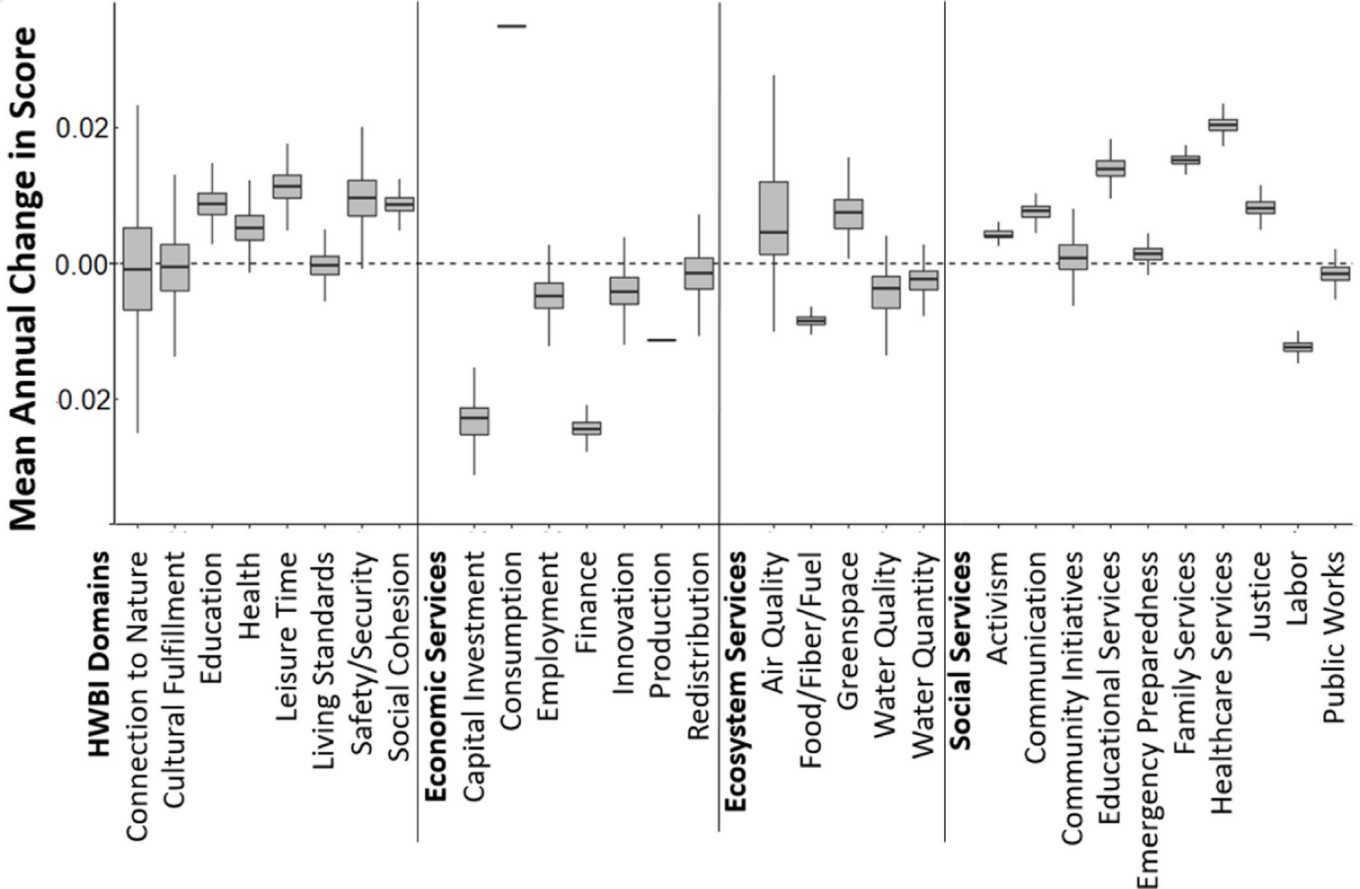
Mean estimated annual change in score from 2000–2017 based on slope over time in each census tract for HWBI domains and services.

Most domains of well-being increased over time, including education, health, leisure time, safety, and social cohesion (Figure 5). Living standards showed very little change over the time period. Downscaled estimates of connection to nature and cultural fulfillment were variable over time, increasing in some census tracts while decreasing in others. The overall increases in well-being corresponded to increases in most social services, with particularly strong increases in educational services, family services, and healthcare services. Labor, and to a small degree public works, tended to decrease over time. Moreover, the majority of economic services decreased during the time period, particularly capital investment and finance. The ecosystem services of food/fiber provisioning, water quality, and water quantity decreased over time in most census tracts, while air quality and greenspace scores typically increased for most census tracts over the time period.

### 3.3. Relationships between Services and Domains

The statistical relationships between domain and service scores over space and time was analyzed using general linear mixed models. For each domain, between 1 and 174 models were identified as top models based on ΔAIC < 4, and included in model averaging to estimate model parameters and overall fit. Services scores explained a substantial portion of variability in most domain scores (cultural fulfillment, R^2^ = 0.45; education, R^2^ = 0.37; health R^2^ = 0.53; leisure time, R^2^ = 0.51; living standards R^2^ = 0.45; safety and security, R^2^ = 0.31; social cohesion, R^2^ = 0.70), with between 1.5–21% of the variability explained by ecosystem services (Appendix A Table A1). To better understand what was driving the patterns, the statistical relationships between domain indicator scores and services scores were examined. Indeed, indicators within a domain were often not consistent in their relationships with services, and regressions at the indicator level often explained greater variability than domain-level regressions (Table 6). However, at the metric level, metrics within an indicator were often strongly correlated and loaded similarly onto the same PC (Appendix A, Tables A2–A5), and metrics within an indicator responded fairly similarly to changes in services (Appendix A Table A6). Therefore, the results here focus on the indicator-level regressions (Table 6) but use the domain and PCA regression results to help aid in interpretation and evaluate the robustness of overall results.

**Figure 6.**
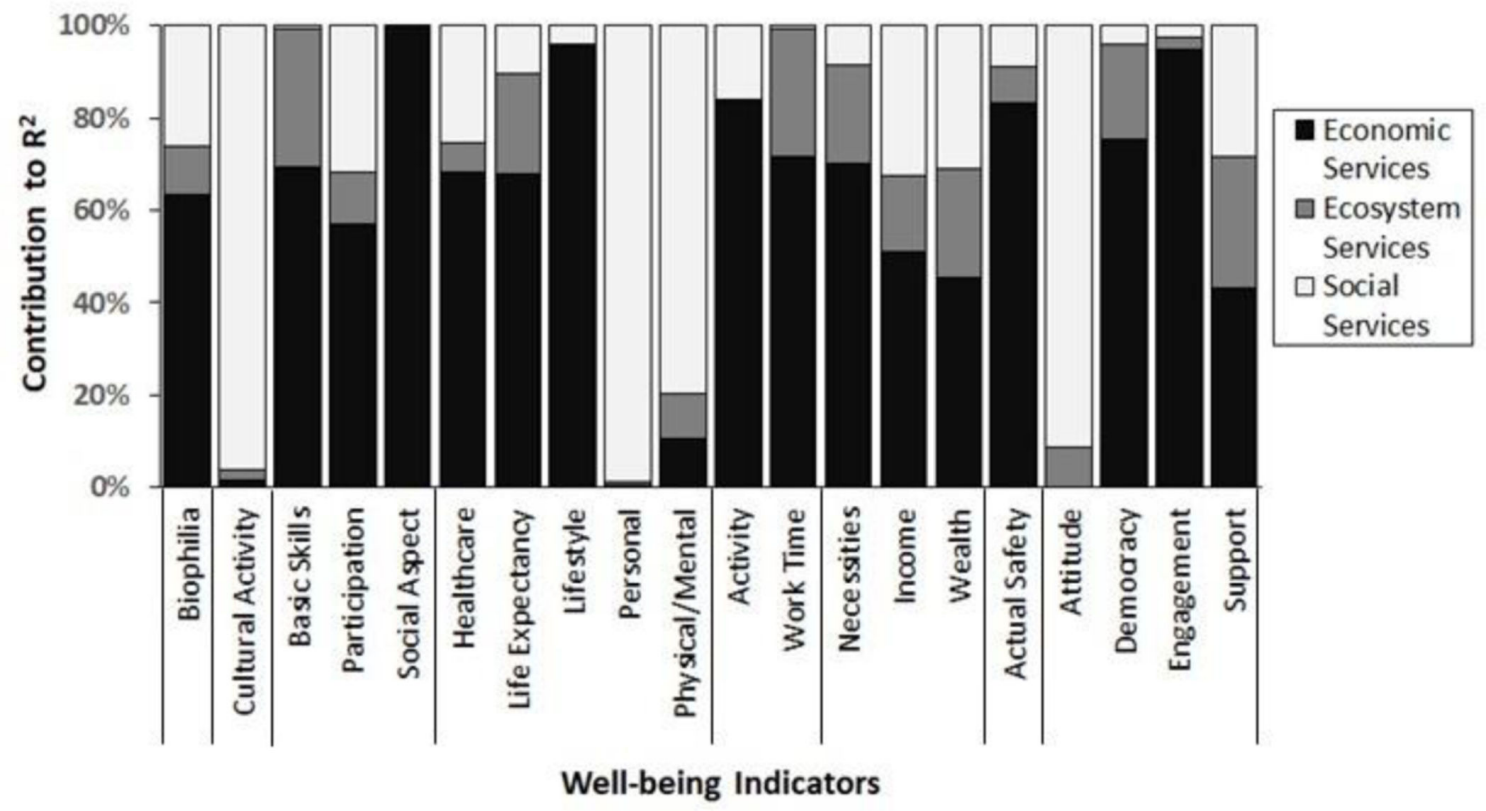
Percent contributions of economic, ecosystem, or social services to explained variability in each well-being indicator based on Shapley scores.

This study calculated Shapley scores for the top set of models (ΔAIC < 4) associated with each domain indicator in order to estimate the percent of variability explained by economic, ecosystem, or social services. Ecosystem services explained as much as 30% of the variability in domain indicator scores (Figure 6). Ecosystem services explained the greatest amount of variability in basic educational skills (30.0%), life expectancy (21.7%), regular work hours (27.9%), basic necessities (21.0%), income (16.6%), wealth (23.4%), democratic engagement (20.6%), and social support (28.3%). Ecosystem services also explained moderate variability in biophilia (10.3%), physical and mental health (9.7%), actual safety (7.9%), and attitude towards community (8.6%). Overall, economic services explained the largest portion of variability across the majority of domain indicators (Figure 6), although social services explained large portions of variability for cultural activities (96.0%), personal well-being (98.6%), physical and mental health (79.7%), and attitude toward community (91.1%).

#### 3.3.1. Connection to Nature

Greenspace was positively and significantly associated with biophilia (Table 6). Food/fiber, specifically the availability of coastal fish and shellfish resources, also had positive associations with biophilia (Appendix A Table A6). Redistribution of wealth explained the greatest amount of variability and was also positively associated with biophilia. PCA results (Appendix A Table A6) suggest this positive relationship with redistribution was primarily driven by the percent of population on social security, while metrics related to increasing inequality (e.g., food stamps) tended to be negatively associated with biophilia. A large number of models (174) were included in the set of top models, with almost all services included in at least one model, but not significant in model averaging (Table 6). Overall, however, services explained very little variability in spatial or temporal patterns of biophilia for Puerto Rico (R^2^ = 0.003).

#### 3.3.2. Cultural Fulfillment

Service scores explained a substantial portion of the spatial and temporal variability in cultural fulfillment (R^2^ = 0.45; Table 6). Cultural activity participation, including arts attendance and belonging to a religious denomination, were predominantly explained by social services (Figure 6). Higher levels of community initiatives were positively associated with higher cultural fulfillment (Table 6). This was also reflected in a positive association between investment/employment in arts with availability of performing arts attendance (Appendix A Table A6). Cultural activity was negatively associated with communication, family services, and healthcare services. In particular, poor physical health measured as high rates of mortality was negatively associated with performing arts attendance (Appendix A Table A6). For ecosystem services, both food/fiber provisioning and water quality were positively associated with cultural activity (Table 6). Greenspace was negatively associated with cultural activity participation (Table 6), primarily driven by a negative association with performing arts attendance in areas with more greenspace (Appendix A Table A6).

#### 3.3.3. Education

A substantial portion of spatial and temporal variability in basic educational skills was explained by ecosystem services (Figure 6), in particular increasing with greenspace but decreasing with provisioning of food and fiber (Table 6). PCA regressions suggested the relationship with food/fiber was largely driven by a positive association between basic skills and land devoted to farmland or forest (Appendix A Table A6). Basic educational skills were also negatively associated with finance services (Table 6), which was strongly negatively correlated with domestic revenues per capita (Appendix A Table A3). Availability and quality of educational services (e.g., education spending and employees per capita) also had a small positive association with basic skills (Table 6). PCA results indicated impacts of some social services on basic skills may have been masked as metrics were aggregated into composite service scores, including negative associations between basic educational skills and either public works spending or public safety spending.

Educational participation and attainment, particularly enrollment and graduation in colleges and universities, was strongly positively associated with finance (Table 6), and in particular with individual savings and loans (Appendix A Table A3). Participation and attainment tended to be higher in areas with less greenspace (Table 6), including those measured as having less forest area or less farmland (Appendix A Table A6), and was positively associated with moderate water quantity (e.g., neither drought nor flood conditions; Table 6). Increases in public safety and emergency spending were negatively associated with participation and attainment (Tables 6, A6).

Social and behavioral aspects of education were predominately explained by economic services (Figure 6). Consumption, measured by higher levels of personal discretionary spending and personal consumption of goods and services, and production, measured by GDP growth was positively associated with social aspects of behavior, including feeling safe at school (Table 6).

#### 3.3.4. Health

Perceptions of care and life expectancy were both primarily explained by economic services, with a moderate portion of variability explained by ecosystem services (Figure 6). Finance scores, represented in part by higher levels of personal loans and lower levels of domestic revenues (Appendix A Table A3), tended to be negatively associated with quality of care and life expectancy (Table 6). Lifestyle, representing healthy behaviors, was also primarily explained by economic services, in particular increasing with higher levels of personal discretionary spending and consumption of goods and services (Table 6). In contrast, personal well-being and physical/mental health were primarily explained by social services (Figure 6), in particular being positively associated with the availability of health and family care services (Table 6).

In aggregate, around 21% of variability in health scores was explained by variability in ecosystem services scores (Appendix A Table A1). Most aspects of health, including care, life expectancy, personal well-being, and physical/mental health, were positively associated with greenspace but negatively associated with provisioning of food and fiber, and to a slight degree air quality as measured by canopy cover (Table 6). PCA regressions suggested the relationships with food/fiber was largely driven by a positive association between disease and mortality rates and land devoted to farmland or forest (Appendix A Table A6). Perceptions of care also had small negative correlations with air quality and water quantity, such that tracts or years with cleaner air or moderate water conditions free from drought or flooding typically had lower rates of hospital visits (Table 6).

#### 3.3.5. Leisure Time

Variability in leisure activity, including physical activity and vacation, was predominately explained by economic services (Figure 6). Leisure activity was strongly positively associated with personal consumption of goods and services and personal discretionary spending (Table 6). A portion of leisure time activity was also positively associated with quality of family services and investment in criminal and environmental justice (Table 6; Appendix A Table A6).

A substantial portion of time available for leisure, measured in part as having regular daytime work hours, was explained by ecosystem services (Figure 6). Available leisure time tended to be positively associated with greenspace but negatively associated with provisioning of food and fiber (Table 6). PCA regressions suggested the relationship with food/fiber was largely driven by a negative association between long work hours and land devoted to farmland or forest (Appendix A Table A6). Available leisure time was also positively associated with water quantity, with tracts or years with extreme water conditions free from drought or flooding typically having greater availability of non-work time (Table 6). Leisure time (non-work hours) was negatively associated with finance, including personal savings and loans.

#### 3.3.6. Living Standards

Spatial and temporal variability in the indicator for work satisfaction, which comprised measures of no fear of job loss and job satisfaction, was not significantly explained by any services variables (fixed R^2^ = 0; total R^2^ = 1.00). The indicator for basic necessities, measured in part as the ratio of income to home cost, was negatively correlated with finance, in particular being negatively associated with personal savings and loans. Ecosystem services explained a moderate amount of variability in basic necessities, with greater home affordability in areas with more greenspace. The food/fiber indicator was negatively associated with the indicator of basic necessities (Table 6), but the composite food/fiber indicator tended to be negatively correlated with presence of farmland and forest, suggesting home affordability was higher in areas with more forest area or farmland (Appendix A Table A6). Basic necessities were also slightly negatively correlated with water quality (Table 6), potentially reflecting negative covariances between clean water and farm or forest area.

Indicators of living standards related to income and wealth were strongly positively correlated with finance (Table 6), in particular being positively associated with personal savings and loans and negatively associated with domestic revenues and public debt (Appendix A Tables A3, A6). A moderate portion of income and wealth were also explained by social services indicators of justice, in particular being positively associated with investment in criminal and environmental justice. Variability in ecosystem services also explained a moderate portion of variability in income and wealth (Figure 6), which tended to be higher in areas with less greenspace (Table 6). Food/fiber indictors were also positively associated with income and wealth (Table 6), but this composite indicator was negatively associated with presence of farmland and forest, suggesting income and wealth were lower in areas with more forest area or farmland (Appendix A Table A6). Income was also positively associated with having moderate water quantity, i.e., years or tracts with neither drought nor flood conditions (Table 6).

#### 3.3.7. Safety and Security

The composite index of safety and security was typically higher in areas or during years of higher consumption, including personal consumption of goods and services and discretionary spending (Table 6). Crime rates, in particular, tended to be associated with higher unemployment and income inequality (Appendix A Table A6). Lower rates of accidental or natural events deaths tended to be associated with increases in production, measured in part by GDP growth (Appendix A Table A6). Safety and security generally were negatively correlated with healthcare services (Table 6), with greater health expenditures and employees in areas or years with higher crime rates (Appendix A Table A6).

In the aggregate, ecosystem services explained only about 7% of variability in safety and security (Figure 6). PCA regressions, however, indicated ecosystem services explained more variability in crime rates (29.3%) than accidental or natural event deaths (<1%; Appendix A Table A6). Lower crime rates were positively associated with food/fiber (Table 6), in particular with measures of agricultural productivity (Appendix A Table A6).

#### 3.3.8. Social Cohesion

The indicator measuring attitude toward one’s community was overwhelming explained by social services (Figure 6), in particular being positively associated with family services, quality and availability of healthcare services, and access to communication technology and information (Table 6). Democratic engagement was negatively associated with the finance indicator (Table 6), which itself was negatively correlated with domestic revenues per capita (Appendix A Table A3), suggesting democratic engagement (e.g., voter turnout) was higher in areas or years with higher domestic revenues (Appendix A Table A6). Social engagement was negatively correlated with levels of personal consumption and production (e.g., GDP growth). To some degree this may reflect greater trust in people and perception that others in the community are helpful, but lower interest in politics, with increasing unemployment and income inequality (Employment PC1, Re-distribution PC1 with Social Cohesion PC1, PC2, PC3; Appendix A Table A6).

The indicator of family bonding, comprised of a single metric, was not significantly explained by any services (fixed R^2^ = 0; total R^2^ = 1). The family bonding metric of time spent watching television was negatively correlated with metrics of social support (Appendix A Table A2). Variability in social support was explained almost equally by economic, ecosystem, and social services (Figure 6). Social support, e.g., having close friends and emotional support, was positively associated with finance, in particular with higher levels of personal savings. To some degree this may reflect lower social support with increasing unemployment and income inequality (Employment PC1, Re-distribution PC1 with Social Cohesion PC1, PC3; Appendix A Table A6). Social support was positively associated with access to communication technology and information (Table 6).

A portion of attitude toward community, democratic engagement, and social engagement was explained by ecosystem services (Figure 6). These three indicators of social cohesion were positively correlated with greenspace (Table 6). Food/fiber tended to be negatively associated with attitude, democracy, and social engagement. However, the composite food/fiber indicator was negatively associated with presence of farmland and forest, suggesting attitude toward community, democratic engagement, and social engagement were higher in areas with more forest area or farmland (Appendix A Table A6). The opposite pattern was seen for the indicator social support, however, with close friends and emotional support tending to be negatively associated with greenspace (Table 6). Attitude toward one’s community and social engagement also had slight negative correlations with air quality, as well as with water quality for attitude (Table 6), possibly reflecting their roles as negative environmental covariates of income inequality which was positively associated with community attitude and engagement.

## 4. Discussion

### 4.1. Relationships between Ecosystem Services and Human Well-Being

This study investigated the potential contributions of indicators of ecosystem services in explaining various components of human well-being in Puerto Rico. Almost all well-being indicators were significantly related to and explained by variability in indicators for greenspace and food/fiber. Greenspace was positively associated with connection to nature, basic education skills, perception of healthcare, life expectancy, personal well-being, physical and mental health, time available for leisure, home affordability, attitude toward community, democratic engagement, and social engagement. These same indicators were usually negatively associated with the composite food/fiber indicator, which comprised metrics of farmland, timber, fishing, and mining. By examining correlated metrics in the PCA-based regressions, this study detected that these negative associations were often driven by a negative correlation between the composite food/fiber indicator and farm area or land/forest available for timber, such that farm and forest area had positive associations similar to those of greenspace, that were otherwise masked by the composite indicator.

Results for Puerto Rico were similar to the U.S. fifty states [16], in that greenspace tended to be positively associated with education, leisure time, and social cohesion. Greenspace has been shown to have positive benefits on education, including test scores, problem solving skills, positive social behaviors, interpersonal skills, and ability to concentrate [10,133–137]. Though higher income is generally associated with increased leisure time, loss of leisure time due to increase work hours has risen in the U.S. since the 1950s [138] and may be especially exacerbated in urban areas with the additional costs of commuting. Greenspace and access to nature have also been shown to promote social behaviors, reduce aggressive behavior, and provide a sense of community and pride [12,139]. In contrast to the Puerto Rico results, the indicator of greenspace for the U.S. fifty states was found to be negatively correlated with connection to nature [16]. The ability to interact with nature may strengthen the appreciation for it [140], and Puerto Rico’s heavily forested landscape and accessible coastline may provide such opportunities.

The positive association between greenspace and human health for Puerto Rico was consistent with previous studies, which have shown positive associations between greenspace and human health outcomes (reviewed in [141]). In San Juan, Puerto Rico, household wealth is not necessarily a predictor of quality of green infrastructure, an opposite pattern to the so-called ‘luxury effect’ observed in many cities [142], and in fact residents with lower socio-economic status actually tend to have more access to green infrastructure [143]. Our results are consistent with prior studies which indicate that, although economic inequalities may be a primary driver of health inequalities, greenspace may have some positive effects on reducing risk [144]. Quality of greenspace is an important consideration, however, as environmental degradation, including issues related to pollution, water quality, and flood risk may exacerbate health risks, and socio-economic status can inhibit abilities of residents to escape or improve such conditions [21,145].

In Puerto Rico, relationships between air quality, water quality, and water quantity and domains of human well-being were generally weaker and less common than those for greenspace. Greenspace provides many of these ecosystem services, through mitigating heat hazards, buffering air-borne or water-borne pollutants, and mitigating drought or flood hazards [141], such that composite measures of greenspace may have already accounted for much of the explained variability. Perceptions of healthcare, measured in part by frequency of hospital visits, tended to be higher in census tracts or years with lower air quality or extreme water quantity in terms of drought or flood conditions. Income, attitude toward community, and social engagement also was typically higher in census tracts with income inequality or receiving public assistance, which also tended to have lower air quality, lower water quality, or extreme water quality in terms of drought or flood conditions. Despite having higher trust in people and government as helpful, these same census tracts, however, were lower in social support, measured as having close friends and emotional support. Lack of extreme water quantity conditions was also positively associated with having more time for leisure time and higher participation and attainment of education. Consistent with results for Puerto Rico, water quantity for the U.S. fifty states [16] was also positively associated with health, leisure time, living standards. Water quality was positively associated with connection to nature in Puerto Rico, consistent with results for the U.S. fifty states [16].

### 4.2. Effects of Economic and Social Services on Human Well-Being

This study assessed the role of ecosystem services in explaining various components of human well-being while accounting for the potential explanatory power of co-occurring economic and social services. In Puerto Rico, variability in indicators of human well-being were predominately explained by economic services, in particular measures of personal finances, including the ability to accumulate personal savings and acquire personal loans, and personal consumption, including personal discretionary spending. Other economic measures that tended to negatively covary with personal finance and consumption, such as measures of income inequality or unemployment, also explained variability for the majority of domains of well-being. Personal consumption is expected to enhance well-being by providing means to obtain basic needs [24], and for Puerto Rico, personal consumption and finances were indeed positively correlated with income and wealth, and were positively associated with aspects of education, healthy lifestyle, leisure activity, and safety. Social engagement, however, tended to be negatively associated with personal consumption and finances and positively associated with redistribution of wealth, a trend seen in the U.S. fifty states as well [16]. Redistribution of wealth has been suggested to improve social cohesion by relieving tensions between different socio-economic classes [146]. In the U.S. fifty states, similar to Puerto Rico, employment was the strongest predictor of living standards and leisure time, but most other domains in the U.S. analysis were more strongly related to social services than economic services [16].

Social services, in particular the quality of family services, healthcare services, and ability to access communication technology and information, explained large amounts of variability in cultural fulfillment, personal well-being, aggregated measures of education, physical and mental health, and attitude toward one’s community in Puerto Rico. This corresponded to similar trends for the U.S. fifty states [16] where family services were positively related to education, health, and social cohesion, and communication was positively associated with cultural fulfillment and health. Family services help societies to combat poverty, violence, and substance abuse, and provide a foundation to ensure communities have opportunities for education, housing, and employment [147], while communication infrastructure promotes public awareness and ensures essential information is communicated [24]. In the U.S. fifty states [16], unlike Puerto Rico, availability and quality of healthcare services was not as strong a predictor of health outcomes. In Puerto Rico, by contrast, increases in accessibility and quality of healthcare services over time was strongly associated with corresponding increases in health outcomes. Additionally for Puerto Rico, increases in availability and quality of educational services were associated with slight positive increases in educational skills, although personal finances were the primary driver of educational participation and attainment, possibly reflecting the essentialness of financial support to optimize educational benefits (e.g., tutoring, extracurricular activities) or allow opportunities for post-secondary education. Quality education is anticipated to have far-reaching impacts beyond educational attainment, including enhancing personal happiness, social responsibility, and national prosperity [24,148]. Though neither strong nor widespread for Puerto Rico, there were small positive associations between educational quality and physical or mental health, basic necessities attainment, and democratic engagement.

Although economic services generally decreased over time, broadly such declines did not translate into declines in composite well-being domain scores for education, health, leisure time, safety, and social cohesion, which tended to increase over time for most census tracts in Puerto Rico. Increases in personal consumption and discretionary spending correlated with some improvements in these domains over time, but they largely corresponded to increases in social services, including increases in family services, healthcare, and communication over the time period, as well as increases in greenspace throughout Puerto Rico over the time period. In contrast, economic services, in particular redistribution of wealth and finance, generally explained patterns of composite well-being over space, in particular correlating with higher connection to nature, higher cultural fulfillment, higher educational participation, and higher living standards. Census tracts that tended to have overall higher well-being throughout the time period also tended to have higher levels of social services, in terms of community initiatives, but lower levels of ecosystem services, with the exception of water quality.

Despite the large explanatory power of economic and social services, however, this analysis detected that portions of well-being could be explained by variability in ecosystem services over space and time. In Puerto Rico, the indicators of ecosystem services explained the greatest amount of variability in the composite index scores for education (16.6%) and health (21%). This exceeded the estimated contribution of the same set of ecosystem services for the same index scores of education and health in the fifty United States, which was less than 10% based on partial R^2^ estimates [16]. For the U.S. fifty states, indicators of ecosystem services were most strongly associated with cultural fulfillment (approximately 27%), leisure time (approximately 55%), and safety (approximately 45%) based on partial R^2^ [16], which for Puerto Rico were explained less than 10% by ecosystem services indicators. Moreover, with the exceptions of leisure time and living standards, social services indicators explained they largest amount of variability in the eight domains of well-being for the fifty U.S. states, ranging from 55 to 85% based on partial R^2^ [16], whereas economic services explained the greatest variability for Puerto Rico. It is important to note that this analysis substituted many surrogate metrics for the US fifty state model specifically chosen and scaled for Puerto Rico [34], was analyzed at the census-tract scale, and used model averaging rather than a single step-wise regression approach to allow broader potential inclusion of explanatory variables, including covarying variables, all of which complicate the ability to compare Puerto Rico results to the U.S. fifty states [16]. Furthermore, by breaking down composite domain scores to indicators, as well as to correlated metrics in this PCA-based analysis, this study was able to detect additional variability explained by ecosystem services that otherwise may have been by masked by the use of aggregated indices.

### 4.3. Modeling Approach and Limitations

This study leveraged the HWBI framework to investigate relationships between changes in ecosystem services and multiple components of well-being because it is a comprehensive approach that simultaneously considers ecosystem, economic, and social components [23,24]. Furthermore, although originally developed for the U.S. fifty states, the HWBI approach is flexible and broadly transferable to any spatial scale, location, or community by customizing metrics within each indicator to suit data availability or unique community needs [23,24], for example, American Indian Alaska Natives [149] or children [150]. The U.S. fifty state HWBI services to domain regression models were built off nationally available county-scale data, to identify broad characterizations of most likely outcomes [16]. By customizing these relationships for Puerto Rico, and at a finer spatial scale, this study was able to examine relationships that better reflect specific local conditions.

A key challenge with modeling relationships between services and domains of well-being, however, is the inconsistency in data availability across relevant spatial scales and time periods. Metrics are often more widely available at the state or county scale, yet smaller spatial scales, such as census tract or housing block, are more likely to be relevant to community or environmental management decisions. This may be particularly true for rural communities, developing countries, or even other U.S. territories, where small-scale data are often scarce and difficult to obtain, or for environmental justice studies that rely on finer scale data to detect social inequities [151]. By leveraging available data to fill in missing data, spatial interpolation approaches such as MERLIN [35,36] can help overcome data limitations and expand opportunities to interpret spatial disparities in well-being, identify decision levers to improve well-being, or monitor trends over time.

Although spatial interpolation enhanced the ability to identify patterns and examine trends in well-being on a neighborhood scale, the reliance on spatial interpolation underscores the need for finer scale data that more accurately capture critical components of well-being, ecosystem services, and socio-economic services. This analysis, and spatial interpolation method, relied heavily on a small subset of metrics available at census tract-scale, which were predominantly measures of employment, education level, and income. Even ecosystem services metrics, which were largely derived from maps of land cover and generally measurable at fine spatial resolutions, suffered from lack of temporal availability. To characterize aesthetic, cultural, spiritual, and recreational ecosystem services, this analysis relied heavily on proxies (e.g., species diversity, greenspace) due to a lack of island-wide data that could more specifically measure how, when, or where ecosystem services are being used. The original U.S. HWBI framework also does not specifically single out aesthetic or cultural ecosystem services as a separate category, primarily due to lack of data, but instead assumes these services can be related to other ecosystem services such as availability of greenspace or water quality [24]. A shift to beneficiary-centric approaches, such as the identification of final ecosystem goods and services [152], can help ensure metrics are identified for future monitoring that more clearly represent the specific attributes of ecosystems that people are using, and can therefore be more directly linked to benefits to well-being.

Though use of proxy metrics and spatial interpolation approaches can help overcome data limitations [35,36], both inherently carry some error, as the interpolation scaling itself depends on statistical relationships between county-scale and tract-scale data and use of proxies assumes underlying correlations between the proxy and ideal metric. The use of composite indicators in the HWBI framework [23] can help to dampen some of the uncertainty associated with individual metrics, as well as detection of spurious relationships among large numbers of metrics, by looking at broad patterns in aggregate. The drawback of using aggregated indicators, however, is greater difficulty in interpreting what specific components of ecosystem, economic, or social services are driving observed patterns. By considering statistical relationships at multiple levels (e.g., metrics vs. aggregated indicators) in addition to composite domain scores (i.e., [16]), this study was better able to evaluate the robustness of relationships and identify which metrics might be driving patterns.

### 4.4. Implications

Connecting environmental decisions to measures of human well-being can help facilitate a discussion of the potential tradeoffs of environmental degradation and potential benefits of ecological interventions and restoration [4,153,154], including providing clearer justifications for investments in natural capital that resonate with people [155–157]. However, well-being measures are still most often linked to economic and social policies, with environmental drivers often being under-considered [158]. Economic and social development, despite having short-term benefits, may threaten health and prosperity in the long-term if societies fail to protect critical natural resources [159,160]. Systems approaches that more fully integrate the three pillars of sustainability (i.e., environmental, economic, and social systems) can help policy makers better assess current condition, provide early warning, formulate decision strategies, and track progress [161].

Puerto Rico has integrated sustainable development goals into land-use planning, but various agencies have competing visions of the degree to which economic investment, revitalization, modern aesthetics, ecological restoration, and livability should dominate future planning [19]. In the aftermath of Hurricane Maria in 2017, there has been greater emphasis on the role of environmental conservation in enhancing livability and mitigating flood risks [20]. Environmental justice issues are of particular concern in Puerto Rico, with over half a million residents, a large percentage of which are below the poverty line, subjected to frequent flooding, exacerbated by effects of wastewater discharges in floodwaters [21,145]. The current study highlights how disparities in the quality and availability of economic and social services in Puerto Rico are associated with disparities in personal well-being, including health, education, basic needs, and safety. However, access to green areas and reductions in flood risk can help to mitigate these disparities, and has the potential to improve well-being and livability across socio-economic classes.

## 5. Conclusions

Linking ecosystem services to multivariate elements of human well-being can serve to complement more traditional community planning or environmental management efforts by facilitating discussion, helping identify potential unintended consequences or under-considered benefits of decisions, helping identify common goals, and identifying areas of uncertainty where more information is needed [162]. Beyond adapting the US HWBI framework to Puerto Rico, this study demonstrates how spatial interpolation approaches expand opportunities to examine human well-being when data are scarce or at spatial scales that may be more relevant to management or environmental justice concerns. In Puerto Rico, although composite measures of well-being were predominantly explained by economic and social disparities, a substantial portion of variability in well-being could be explained by quality and quantity of ecosystem services. An examination of the potential benefits of ecosystem services, within the context of social and economic conditions, can help ensure that key well-being objectives and creative alternatives to achieve them are not overlooked.

## Supplementary Material

1

2

3

4

5

6

7

8

9

10

## Figures and Tables

**Table 1.** Metrics used to describe each indicator within each HWBI domain [modified from 34, 36]. Number of years of available data from 2000–2017 is given in parentheses; data sources are provided as footnotes. Metrics in bold were available at census tract scale. Metrics in italics were inverted to maintain a positive relationship with well-being. Superscripts give reference numbers for data sources.

WELL-BEING DOMAIN: Indicator: Metrics
CONNECTION TO NATURE: Biophilia: Connection to all of life (1) [37]

CULTURAL FULFILLMENT: Cultural Activity: Performing arts attendance (1) [38]; Belonging to religious denomination (1) [37]

EDUCATION: Basic Skills of Youth: Standard math test achievement (2) [39]; Standard reading test achievement (2) [39]; Standard science test achievement (2) [39]
Participation/Attainment: *Adult illiteracy rate* (3) [40]; **High school graduation rate** (14) [32]; **Post-secondary education enrollment** (14) [32]; **Post-secondary education graduation** (14) [32]
Social Aspects: *Children feeling unsafe at school* (5) [41]; Children’s health (4) [42]; Children’s social behavior (5) [41]

HEALTH: Care: Regular doctor visits (17) [43]; Satisfaction with hospital care (2) [43]
Life Expectancy/Mortality: *Asthma mortality rate* (16) [44]; *Cancer mortality rate* (16) [44]; *Diabetes mortality rate* (16) [44]; *Heart disease mortality rate* (16) [44]; *Infant mortality rate* (18) [44]; Life expectancy at birth (18) [45]; *Suicide mortality rate* (16) [44]
Personal Well-Being: Happiness (1) [37]; Life satisfaction (6) [43]; Perceived health (18) [43]
Lifestyle/Behavior: *Alcoholic beverage consumption* (17) [43]; Healthy Behaviors Index (9) [43]; *Teen pregnancy rate* (18) [43]; *Teen smoking rate* (4) [41]
Physical/Mental Conditions: *Lifetime adult asthma rate* (18) [43]; *Lifetime adult cancer rate* (8) [43]; *Lifetime child asthma rate* (13) [43]; *Lifetime adult depression rate* (9) [43]; *Lifetime adult diabetes rate* (18) [43]; *Lifetime adult heart attack rate* (14) [43]; *Lifetime adult heart disease rate* (14) [43]; *Adult obesity rate* (18) [43]; *Lifetime adult stroke rate* (14) [43]

LEISURE TIME: Leisure Activity: Physical activity participation (18) [43]; Time spent on vacation (10) [46]
Time Spent: Time spent on leisure or relaxing (1) [47]
Working Age Adults: *Percent working long work hours* (12) [48]; Percent daytime work hours (12) [48]

LIVING STANDARDS: Basic Necessities: Food security (1) [49]; ***Home ownership cost/income ratio*** (13) [32]
Income: **Median household income** (13) [32]; ***Poverty rate*** (13) [32]; ***Persistent poverty rate*** (11) [32]
Wealth: **Median home value** (13) [32]; ***Mortgage debt*** (13) [32]
Work: No fear of job loss (13) [50]; Job satisfaction (1) [51]

SAFETY/SECURITY: Actual Safety: *Accidental death rate* (16) [44]; *Natural event injury/death rate* (15) [52]; *Millions of dollars in natural event damage* (17) [52]; *Property crime rate* (17) [53]; *Violent crime rate* (17) [53]
Perceived Safety: Perceived safety (1) [54]
Risk: *Social Vulnerability Index* (1) [55]

SOCIAL COHESION: Attitude Toward Community: Trust in people (1) [37]; City satisfaction (6) [56]; Feeling close to one’s town or city (1) [57]; Perception that others are helpful (1) [37]
Democratic Engagement: Interest in politics (1) [37]; Registered voters (4) [58]; Satisfaction with democracy (1) [37]; Trust in government (1) [37]; Voice in government (1) [37]; Voter turnout (4) [58]
Family Bonding: *Time spent watching television* (5) [41]
Social Engagement: Child organized activity participation (5) [41]; Participation in organized group (1) [35]; Volunteer rate (1) [35]
Social Support: Having close family or friends (1) [35]; Getting emotional or social support (6) [43]

**Table 2.** Metrics used to describe indicators (underlined) for each Economic service, with available years of data (2000–2017) in parentheses. Metrics available at census tract scale are in bold. Italics indicate metrics that were inverted to maintain a positive relationship with service provisioning.

ECONOMIC SERVICE: Indicator: Metrics

CAPITAL INVESTMENT: Capital Formation: Per capita domestic investment (18) [59]; Commercial Durables: Yearly change in private inventories (10) [60]; New Housing Starts: **New housing units per capita** (18) [61]; Infrastructure Investments: Per capita investment in private equipment (10) [60]; Per capita in private construction (10) [60]; Per capita investment in public equipment (10) [60]; Per capita in public construction (10) [60]

CONSUMPTION: Cost of Living: Average consumer price index (18) [59]; Discretionary Spending: Personal discretionary spending (10) [60]; Goods and Services: Personal durable goods consumption (10) [60]; Personal non-durable goods consumption (10) [60]; Personal services consumption (10) [60]; Sustainable Consumption: Per capita spending on agricultural marketing (11) [62]

EMPLOYMENT: Employment: **Percent employed** (14) [32]; **Manufacturing employees per capita** (10) [32]; Manufacturing employees per capita (18) [63]; Percent self-employed (10) [64]; Employment Diversity: ***Ogive Index*** (10) [32]; Unemployment: ***Percent unemployed*** (14) [32]

FINANCE: Governance: Total domestic revenues per capita (9) [65]; *Gross public debt per capita* (11) [66]; Loans: Commercial loans per capita (9) [65]; Individual loans per capita (9) [65]; Real estate loans per capita (9) [65]; Savings: Personal savings per capita (9) [65]; **Finance employees per capita** (10) [32]

INNOVATION: Investment: Research investment per capita (11) [67]; Patents and Products: Patents per capita (16) [68]
PRODUCTION: Exports: Exports as percent GDP (18) [69]; Household Services: Volunteer participation per capita (14) [70]; Market Goods and Services: Yearly change in GDP (18) [59]; Sustainable Production: Expenditures on renewables per capita (17) [71]

RE-DISTRIBUTION: Income Equality: **GINI Index** (12) [32]; Public Support: Childcare funding per capita (15) [72]; ***Percent population using food stamps*** (9) [32]; **Percent population on social security** (10) [32]; ***Percent population receiving cash assistance*** (9) [32]

**Table 3.** Metrics used to describe indicators (underlined) for each Ecosystem service, with available years of data (2000–2017) in parentheses. Metrics available at census tract scale are in bold. Italics indicate metrics that were inverted to maintain a positive relationship with service provisioning.

ECOSYSTEM SERVICE: Indicator: Metrics

AIR QUALITY: Usable Air: Percent canopy cover (air pollutant removal) (2) [73–75]; Percent good Air Quality Index (18) [76]; **Percent canopy cover in developed space** (**urban heat reduction**) (2) [73–75,77]

FOOD/FIBER/FUEL PROVISIONING: Food and Fiber: **Crop production index** (1) [78]; Percent farmland (4) [79]; Total factor productivity (14) [80]; Fish and shellfish landings (5) [81]; Food production index (17) [82]; **Percent prime farmland** (1) [78]; Land area suited for forestry (1) [83]; **Percent forest cover** (2) [74,75]; Raw Materials: Metric tons of clay, salt, stone mining (16) [84]

GREENSPACE: Natural Areas: **Percent natural area** (2) [74,75]; Annual visitors to national parks (18) [85]; Recreation/Aesthetics: **Species richness** (1) [86]; **Species richness** (2) [74,75,86]; **Water area per capita** (2) [87]

WATER QUALITY: Usable Water: Percent good beach days (17) [88]; **Percent waterbodies assessed as good** (7) [89]; ***Relative erosion potential*** (1) [90]

WATER QUANTITY: Available Water: **Percent area not drought vulnerable** (1) [78]; **Percent area not in flood zone** (1) [91]; *Number of flood events* (18) [92]; Percent days with no drought risk (18) [93]; **Available water storage** (1) [78]

**Table 4.** Metrics used to describe indicators (underlined) for each Social service, with available years of data (2000–2017) in parentheses. Metrics available at census tract scale are in bold. Italics indicate metrics that were inverted to maintain a positive relationship with service provisioning.

SOCIAL SERVICE: Indicator: Metrics

ACTIVISM: Participation: Individual donations to political organizations (18) [94]; **Religious/civic employees per capita** (14) [32];

COMMUNICATION: Accessibility: Percent population with cell phone subscription (18) [69]; **Percent households with telephone** (14) [32]; Industry Infrastructure: Percent households with broadband (17) [69]; Providers: Information employees per capita (18) [63]; **Information employees per capita** (10) [32]; Public Services Communication: Number of promotion projects (10) [65]; Federal spending per capita on public broadcasting (11) [62]; Spending per capita on public broadcasting (14) [95]; Quality: Voice and Accountability Index (18) [96]

COMMUNITY-/FAITH-BASED INITIATIVES: Investment: **Arts/culture employees per capita** (10) [32]; Arts/culture employees per capita (18) [63]; Federal spending per capita on arts/museums (11) [62]; Spending per capita on film industry (14) [95]; Providers: Non-profits per capita (1) [97]

EDUCATIONAL SERVICES: Accessibility: Percent schools with alternate education (17) [98]; Percent students receiving financial aid (14) [99]; Public schools per capita (17) [100]; Confidence: Cumulative Promotion Index (17) [100,101]; Investment: Education expenditures per capita (14) [95]; Federal education spending per capita (11) [62]; Per pupil spending (16) [100]; Providers: Education employees per capita (18) [63]; **Education, Health, Social employees per capita** (10) [32]; Teachers per capita (17) [100]; **Education/training/library employees per capita** (10) [102]; *Student/teacher ratio* (17) [100]

EMERGENCY PREPAREDNESS: Post-Disaster Response: Public Health Emergency Preparedness funding per capita (10) [103]; Dollars per flood claim (16) [104]; Federal disaster response spending per capita (11) [62]; Pre-Planning Disaster: Flood insurance policies per capita (8) [105]; Insurance employees per capita (18) [63]; **Insurance and finance employees per capita** (10) [32]; Federal non-disaster emergency management spending per capita (11) [62]; Responders: Emergency employees per capita (18) [63]; Public safety budget per capita (14) [95]; **Protective service employees per capita** (10) [102];

FAMILY SERVICES: Accessibility: *Days to child services* (8) [106]; Percent homeless with shelter (13) [107]; Effectiveness: Percent adoptions taking less than one year (16) [108]; Rate of preventative services (12) [109]; Percent without a maltreatment recurrence (10) [109]; Investment: Federal spending on child welfare per capita (11) [62]; Spending on public welfare per capita (14) [95]; Providers: Family services employees per capita (18) [63]; Family services employees per child (12) [109]; **Education, Health, and Social services employees per capita** (10) [32]; **Community and social services employees per capita** (10) [102]

HEALTHCARE SERVICE: Accessibility: Health facilities per capita (1) [110]; **Percent insured** (10) [32]; *Health Professional Shortage Areas Score* (1) [110]; Medicare enrollees per capita (18) [111]; **Percent population with Medicare** (9) [32]; Investment: Federal spending on health and human services per capita (11) [62]; Health expenditures per capita (14) [95]; Providers: Health employees per capita (18) [63]; **Health, Education, or Social employees per capita** (10) [32]; **Health practitioners or support per capita** (10) [102]; Quality: Percent treated properly for heart attack (10) [112]; Percent treated properly for heart failure (11) [112]; Percent treated properly for pneumonia (11) [112]; Percent treated properly for surgical infection (11) [112]

JUSTICE: Accessibility: Clearance rate of appellate cases (15) [113]; Clearance rate of trial cases (8) [113]; Environmental: Federal environmental grants per capita (15) [114]; ***Population within 1000 m of toxic release facilities*** (18) [115]; Investment: Federal justice spending per capita (11) [62]; Justice and public safety spending per capita (14) [95]; Federal environmental financial aid per capita (11) [62]; Environmental spending per capita (14) [95]; Providers: Justice employees per capita (18) [63]; **Government employees per capita** (10) [32]; **Legal service employees per capita** (10) [102]; Quality: Percent convictions in criminal cases (12) [113]; Percent convictions in criminal cases (8) [113]; Rule of Law Index (18) [116]

LABOR: Confidence: Federal labor financial aid per capita (11) [62]; Effectiveness: **Rate of non-injuries to workers** (10) [117]; Rate of non-injuries to workers (18) [117]; Employee Rights: Percent employees not filing Equal Employment Opportunity charges (9) [32,118]

PUBLIC WORKS: Accessibility**: Percent using public transportation** (14) [32]; *Water quality violations per facility* (18) [119]; Investment: Federal transportation financial aid per capita (11) [62]; Bus and maritime spending per capita (14) [95]; Federal environmental financial aid per capita (11) [62]; National park spending per capita (14) [95]; Federal highway financial aid per capita (11) [62]; Highway spending per capita (14) [95]; Federal telecommunications financial aid per capita (11) [62]; Public utility spending per capita (14) [95]; Providers: Utility and transportation employees per capita (18) [63]; **Utility and transportation employees per capita** (10) [32]; Quality: Percent of bridges with no structural deficiencies (18) [120]; Percent of landfill waste diverted (12) [121]; Recycling facilities per capita (1) [121]; Percent of time not under outage (18) [122]; Fraction of roads in good condition (11) [123]; Quantity: Airport passengers per year (13) [124]; Percent of bridges not functionally obsolete (18) [120]; *Traffic per km of road* (13) [125]

**Table 5.** Diagnostic statistics (regression R^2^) from down-scaling Step 1, which relates tract-scale values to county-scale values for metrics available at tract scale, and Step 2, which uses metrics available at the tract scale to predict metrics available at the county scale. Predictive accuracy of down-scaling was evaluated by examining correlations (mean Pearson’s *r*) between known tract-scale values and predicted tract-scale values for metrics available at the tract-scale, and between known county-scale values and predicted county-scale values for all metrics. Well-being domain diagnostics were derived from the analysis in [36] for comparison with service diagnostics developed here.

		Step 1: Tract Set 1 vs. County Set 1	Step 2: County Set 1 vs. County Set 2		Accuracy: Known vs. Predicted	

	Total Metrics (# Tract-scale)	Mean R^2^	Mean # of Predictor Variables	Mean R^2^	Mean *r* (Tract-Scale)	Mean *r* (County-Scale)

**Well-Being Domains**						
Connect. to Nature	1 (0)	—	7.0	0.41	—	1.00
Cultural Fulfill.	2 (0)	—	7.0	0.30	—	1.00
Education	9 (3)	0.48	1.9	0.71	0.96	0.89
Health	25 (0)	—	5.1	0.74	—	1.00
Leisure Time	4 (0)	—	4.3	0.68	—	1.00
Living Standards	7 (6)	0.64	1.1	0.99	0.88	0.72
Safety/Security	5 (0)	—	4.8	0.94	—	1.00
Social Cohesion	15 (0)	—	5.9	0.64	—	1.00

**Economic Services**						
Capital Invest.	7 (1)	0.37	5.4	1.00	0.94	0.94
Consumption	6 (0)	—	6.3	1.00	—	1.00
Employment	6 (4)	0.82	2.8	1.00	0.89	0.87
Finance	7 (1)	0.95	6.6	1.00	0.99	1.00
Innovation	2 (0)	—	6.5	0.56	—	0.97
Production	4 (0)	—	4.8	1.00	—	1.00
Re Distribution	5 (4)	0.86	2.4	1.00	0.97	0.93

**Ecosystem Services**						
Air Quality	2 (1)	1.00	3.5	0.80	1.00	0.98
Food/Fiber/Fuel	10 (4)	1.00	5.1	0.79	1.00	0.99
Greenspace	5 (4)	1.00	2.2	1.00	0.97	0.99
Water Quality	3 (2)	0.97	4.0	0.86	0.87	0.83
Water Quantity	5 (3)	1.00	4.6	0.85	0.88	0.92

**Social Services**						
Activism	2 (1)	0.89	4.0	1.00	0.88	0.92
Communication	9 (2)	0.94	4.4	1.00	0.83	0.96
Community Initiatives	5 (1)	0.94	5.0	0.97	0.88	0.95
Education	12 (2)	0.88	6.6	0.77	0.96	0.93
Emergency Prep.	10 (2)	0.92	4.5	1.00	0.95	0.98
Family Services	11 (2)	0.86	3.7	1.00	0.97	0.98
Healthcare	14 (4)	0.90	5.6	0.77	0.92	0.93
Justice	14 (3)	0.96	4.6	0.90	0.89	0.96
Labor	4 (1)	0.97	5.0	1.00	0.86	0.96
Public Works	20 (2)	0.94	5.5	0.87	0.89	0.95

**Table 6.** Estimated model parameters from full model averaging for each well-being indicator (columns) as a function of services (rows). Empty cells indicate the variable was not included in any of the top models by model selection. Shading indicates percent contribution of each variable to fixed R^2^, estimated by Shapley scores, as described in key.

	Key to Shading	Education					Health				Leisure			Living Standard			Social Cohesion				
		
	1–10% of R^2^	Biophilia	Cultural Activity	Basic Skills	Participation	Social Aspect	Care	Life Expect.	Lifestyle	Personal	Physical/Mental	Activity	Work Time	Necessities	Income	Wealth	Actual Safety	Attitude	Democracy	Engagement	Support
	10–20% of R^2^																				
	20–30% of R^2^																				
	30–40% of R^2^																				
	>40% of R^2^																				
	>60% of R^2^																				

**Number of Models**		174	2	1	1	26	10	8	35	4	14	10	1	7	1	4	15	6	3	32	0
**R^2^ Fixed**		0.003	0.45	0.44	0.43	0.42	0.34	0.55	0.70	0.48	0.38	0.51	0.27	0.46	0.48	0.48	0.31	0.55	0.45	0.35	0.36
**R^2^ Total**		0.13	0.95	0.96	0.98	1.00	0.93	0.68	0.91	0.94	0.87	0.95	0.76	0.93	0.98	0.98	0.84	0.90	0.96	0.75	0.90

**Intercept**		0.41 ***	1.36 ***	1.26 ***	−0.73 ***	−0.68 *	1.21 ***	0.63 ***	0.17 ***	−0.96 ***	0.13 ***	−0.64 ***	0.65 ***	1.20 ***	−1.08 ***	−1.21 ***	0.80 ***	−0.02 ^NS^	1.02 ***	1.40 ***	0.03 ^NS^

**Economic**	**Capital Invest.**	0.00 ^NS^		−0.08 ***	0.14 ***	0.00 ^NS^	−0.08 ***	−0.15 ***	0.00 ^NS^		−0.10 ***	−0.03*	−0.07 ***	−0.61 ***	0.17 ***	0.14 ***	0.01 ^NS^	0.00 ^NS^	−0.14 ***	−0.09 ***	0.12 ***
	**Consumption**	0.00 ^NS^				**0.80** ***			**0.31** ***			**0.59** ***					0.29 *			**−0.66** ***	
	**Employment**	−0.06 ***				0.01 ***			0.06 ***		0.00 ^NS^						−0.08 ***			0.13 ***	−0.17 ***
	**Finance**	0.00 ^NS^		**−1.13** ***	1.83 ***	−0.02 ***	**−0.64** ***	−0.16 ***				0.35 ***	**−0.20** ***	−0.96 ***	2.37 ***	2.37 ***	0.07**		**−0.85** ***	−0.17 ***	0.39 ***
	**Innovation**	−0.02*	0.12 ***	−0.05 ***	0.23 ***	0.00 ^NS^		−0.04 ***		−0.05**	0.00 ^NS^	0.04 ***	−0.04 ***	−0.01 ^NS^	0.18 ***	0.31 ***	0.07 ***	−0.04 ***	−0.03 ***	−0.09 ***	0.07 ***
	**Production**	−0.01 ^NS^				1.42**											−0.38 ^NS^			−0.92 ***	
	**Redistribution**	0.10 ***	0.16 ***	0.08 ***					0.01 ^NS^	0.10 ***	0.08 ***	0.04 ***		0.05**	−0.14 ***			0.04 ***	0.07 ***		

**Ecosystem**	**Air Quality**	0.00 ^NS^				0.00 ^NS^	−0.03 ***	−0.01 **		0.01 ^NS^		0.00 ^NS^				0.00 ^NS^	0.01**	−0.02 ***		−0.03 ***	0.03 ***
	**Food/Fiber/Fuel**	0.00 ^NS^	0.12**	−0.51 ***	0.09 **		−0.08 **	−0.12 ***	−0.03 ^NS^	−0.24 ***	−0.11 ***	0.03 ^NS^	−0.12 ***	−0.21 ***	0.33 ***	0.54 ***	0.20 ***	−0.29 ***	−0.19 ***	−0.17 ***	0.31 ***
	**Greenspace**	0.02 ^@^	−0.01 ^@^	0.16 ***	−0.07 ***	0.00 ^NS^	0.08 ***	0.08 ***	0.00 ^NS^	0.09 ***	0.07 ***	0.00 ^NS^	0.04 ***	0.21 ***	−0.06 ***	−0.20 ***	−0.08 ***	0.12 ***	0.06 ***	0.15 ***	−0.18 ***
	**Water Quality**	0.02 ^NS^	0.09 ***			−0.005 ***					0.00 ^NS^			−0.04**				−0.02*			
	**Water Quantity**	0.00 ^NS^			0.13 ***	−0.01 ***	−0.07 ***	0.01 ^NS^	0.03 ***	−0.01 ^NS^		0.00 ^NS^	0.04 ***		0.09 ***		0.00 ^NS^				

**Social**	**Activism**	0.00 ^NS^				0.00 ^NS^		0.00 ^NS^						0.00 ^NS^						0.00 ^NS^	0.02 ^NS^
	**Communication**	0.00 ^NS^	−0.65 ***			0.00 ^NS^		0.12 ***	0.00 ^NS^	0.85 ***	0.22 ***	0.41 ***					−0.16 ***	0.28 ***		−0.05 ^NS^	0.44 ***
	**Community Init.**	0.00 ^NS^	0.12 ***			−0.01 ***	0.00 ^NS^	0.09 ***	0.01 ^NS^	−0.14 ***			0.04 ***	0.31 ***	0.09 ***				0.00 ^NS^	0.10 ***	
	**Educational**	0.00 ^NS^		0.06*		0.00 ^NS^	0.00 ^NS^	0.01 ^NS^			0.02 ^NS^	−0.02 ^NS^		0.19 ***	−0.09**	−0.03 ^NS^			0.09 ***	−0.11 ***	
	**Emergency Prep.**	0.00 ^NS^			−0.13 ***	0.02 ***	0.00 ^NS^		0.05**							−0.56 ***				0.04 ^NS^	−0.06 ^NS^
	**Family Service**	0.00 ^NS^	−0.86 ***			0.03 ***			0.13 ***	0.66 ***		0.39 ***					0.00 ^NS^	0.23 ***		−0.05 ^NS^	
	**Healthcare**	−0.01 ^NS^	−0.70 ***			0.03 ***			0.07 ***	1.10 ***	0.34 ***						−0.28 ***	0.51 ***		0.17 ***	−0.15 ***
	**Justice**	0.00 ^NS^							0.00 ^NS^	0.38 ***	0.22 ***	0.16 ***		0.00 ^NS^			0.01 ^NS^	0.02 ^NS^		−0.11 **	
	**Labor**	0.00 ^NS^				0.01^NS^	−0.26 ***		0.00 ^NS^			0.18 ***			0.33 ***	0.51 ***	−0.15 ***			0.04 ^NS^	0.17 **
	**Public Works**	0.11 **				−0.01*	0.04 ^NS^	−0.06 **						−0.27 ***	−0.23 ***			0.00 ^NS^	0.00 ^NS^		0.02 ^NS^

Variable significance indicated as

*** *p* < 0.001

** 0.001 < *p* <0.01

* 0.01 < *p* < 0.05

@ 0.05 < *p* < 0.1

NS, not significant.

## APPENDIX

### Appendix A

**Table A1.** Estimated model parameters from full model averaging for each human well-being Domain (columns) as a function of Services. Empty cells indicate a variable was not included in any of the top models by model selection. Shading indicates percent contribution of each variable to fixed R2, estimated by Shapley scores, as described in key

	Key to Shading	Connection Nature	Cultural Fulfillment	Education	Health	Leisure	Living Standards	Safety and Security	Social Cohesion
	1–10% of R^2^								
	10–20% of R^2^								
	20–30% of R^2^								
	30–40% of R^2^								
	>40% of R^2^								
	>60% of R^2^								

**Number of Models**		174	2	5	44	71	1	15	37
**R^2^ Fixed**		0.003	0.45	0.37	0.53	0.51	0.45	0.31	0.70
**R^2^ Total**		0.13	0.95	0.92	0.76	0.86	0.98	0.84	0.90
**% Contribution of**									
**Economic Services**		63.5	1.7	0.5	9.2	74.9	56.3	83.2	97.5
**Ecosystem Services**		10.3	2.3	16.6	21.0	5.5	6.6	7.9	1.5
**Social Services**		26.2	96.0	82.9	69.9	19.6	37.1	8.9	1.0

**Intercept**		0.41 ***	1.36 ***	0.17 ***	0.42 ***	0.04 ^NS^	−0.16 ^NS^	0.8 ***	0.36 ***

**Economic Services**	**Capital Investment**	0.00 ^NS^			−0.06 ***	−0.03 *		0.01 ^NS^	
	**Consumption**	0.00 ^NS^				0.11 ^@^		0.29*	**0.22** ***
	**Employment**	−0.06 ***						−0.08 ***	−0.03 ***
	**Finance**	0.00 ^NS^					0.95 ***	0.07**	−0.03**
	**Innovation**	−0.02*	0.12 ***		0.00 ^NS^	0.00 ^NS^	0.11 ***	0.07 ***	−0.01*
	**Production**	−0.01 ^NS^				0.13 ^NS^		−0.38 ^NS^	
	**Redistribution**	0.1 ***	0.16 ***	0.08 ***	0.02 ***	0.02 ***			0.01 ***

**Ecosystem Services**	**Air Quality**	0.00 ^NS^			0.00 ^NS^	0.02 ***	0.02******	0.01**	
	**Food/Fiber/Fuel**	0.00 ^NS^	0.12**	−0.17 ***	−0.12 ***	−0.09 ***	0.12 ***	0.20 ***	−0.04 ***
	**Greenspace**	0.02 ^@^	−0.01 ^@^	0.03 ***	0.06 ***	0.00 ^NS^	−0.03 ***	−0.08 ***	0.02 ***
	**Water Quality**	0.02 ^NS^	0.09 ***	−0.03 ***	−0.01 ^NS^	0.00 ^NS^			0.00 ^NS^
	**Water Quantity**	0.00 ^NS^		0.02*	0.00 ^NS^	0.00 ^NS^	0.04 ***	0.00 ^NS^	0.00 ^NS^

**Social Services**	**Activism**	0.00 ^NS^							0.01*
	**Communication**	0.00 ^NS^	−0.65 ***	0.17 ***	0.11 ***	0.18 ***		−0.16 ***	0.00 ^NS^
	**Community Initiatives**	0.00 ^NS^	0.12 ***			−0.02 *	0.08 ***		0.03 ***
	**Educational Services**	0.00 ^NS^		0.00 ^NS^	0.01 ^NS^				0.03 ***
	**Emergency Prepared**	0.00 ^NS^				0.01 ^NS^	−0.17 ***		−0.02 ^NS^
	**Family Services**	0.00 ^NS^	−0.86 ***	0.15 ***	0.00 ^NS^	0.06 *		0.00 ^NS^	−0.08 ***
	**Healthcare Service**	−0.01 ^NS^	−0.70 ***	0.27 ***	0.20 ***	0.26 ***		−0.28 ***	0.00 ^NS^
	**Justice**	0.00 ^NS^		0.03 ^NS^	0.05 ***	0.05 **		0.01 ^NS^	−0.01 ^NS^
	**Labor**	0.00 ^NS^				0.13 ***	0.33 ***	−0.15 ***	0.10 ***
	**Public Works**	0.11 **		−0.03 *	−0.05 ***	0.00 ^NS^	−0.18 ***		

Variable significance indicated as

*** *p* < 0.001

** 0.001 < *p* < 0.01

* 0.01 < *p* < 0.05

@ 0.05 < *p* < 0.1

NS, not significant.

**Table A2.** Metrics contributing most strongly to each principal component (PC) for well-being domains, with metrics available at census-tract scale in bold, standardized loadings with that PC in parentheses, and italics indicating negative correlation with PC. Indicator categories for each metric from the original HWBI (Smith et al. 2012) are underlined

Domain PC Variance Explained	Metrics

Connection Nature	
PC1: 1.00	Biophilia: Connection to all of life (1.00)

Culture Fulfillment	
PC1: 0.50	Participation: *Performing arts attendance (−0.71)*; Belonging to religious denomination (0.71)

Education	
PC1: 0.37	Basic Skills: Standard math test achievement (0.94); Standard reading test achievement (0.95); Standard science test achievement (0.95)
PC2: 0.24	Attainment: **High school graduation rate** (0.79); **Post-secondary education enrollment** (0.89); **Post-secondary education graduation** (0.82)
PC3: 0.17	Social Development: Children feeling unsafe at school (0.26); *Children’s positive social behavior (−0.92)*; Attainment: Adult illiteracy rate (0.76)

Health	
PC1: 0.17	Care: Regular doctor visits (0.47); *Satisfaction with hospital care (−0.33)*; Life Expectancy/Mortality: *Asthma mortality rate (−0.49)*; *Infant mortality rate (−0.65);* Life expectancy at birth (0.89); Lifestyle/Behavior: Healthy Behaviors Index (0.36); *Teen pregnancy rate (−0.37); Teen smoking rate (−0.80*; Physical/Mental Conditions: *Lifetime child asthma rate (−0.55)*; Lifetime adult diabetes rate (0.49); Adult obesity rate (0.60)
PC2: 0.13	Personal Well-Being: *Happiness (−0.81); Life satisfaction (−0.51)*; *Perceived health (−0.51)*; Physical/Mental Conditions: Lifetime adult asthma rate (0.31); Lifetime adult cancer rate (0.20); Lifetime adult depression rate (0.80);Lifetime adult heart attack rate (0.53); Lifetime adult heart disease rate (0.73); Lifetime adult stroke rate (0.50)
PC3: 0.12	Life Expectancy/Mortality: *Cancer mortality rate (−0.64);* Diabetes mortality rate (0.72); Heart disease mortality rate (0.76); Suicide mortality rate (0.72); Lifestyle/Behavior: *Alcoholic beverage consumption (−0.31)*

Leisure Time	
PC1: 0.47	Participation: Physical activity participation (0.68); Work: Long work hours (0.84); Regular daytime work hours (0.84)
PC2: 0.26	Participation: Time spent on vacation (0.98)

Living Standards	
PC1: 0.43	Income: **Median household income** (0.91); ***Poverty rate*** *(−0.84)*; ***Persistent poverty rate*** *(−0.45)*; Wealth: **Median home value** (0.80); ***Mortgage debt*** *(−0.66);* Basic Necessities: **Home ownership cost/income ratio** (0.44);
PC2: 0.14	Work: No fear of job loss (1.0)

Safety/Security	
PC1: 0.39	Actual Safety: Property crime rate (0.97); Violent crime rate (0.96)
PC2: 0.29	Actual Safety: Accidental death rate (0.70); Natural event injury and death rate (0.62); *Millions of dollars in natural event damage (−0.76)*;

Social Cohesion	
PC1: 0.30	Engagement: Participation in organized group (0.95); Volunteer rate (0.95); Community: Perception that others are helpful (0.96); Support: *Having close family or friends (−0.87);* Democracy: *Interest in politics (−0.82)*; *Satisfaction with democracy (−0.45)*;
PC2: 0.18	Democracy: Trust in government (0.90); Voice in government (0.85); Community: Trust in people (0.89);
PC3: 0.17	Bonding: Time spent watching television (0.71); Support: *Getting emotional or social support (−0.73);* Community: *City satisfaction (−0.47)*; Democracy: Registered voters (0.52); Voter turnout (0.73); Engagement: Child organized activity participation (0.63);

**Table A3.** Metrics contributing most strongly to each principal component (PC) for Economic Services, with metrics available at census-tract scale in bold, standardized loadings with that PC in parentheses, and italics indicating negative correlation with PC. Indicator categories for each metric from the original HWBI (Smith et al. 2012) are underlined

Service PC Variance Explained	Metrics

Capital Investment	
PC1: 0.52	Capital Formation: Per capita domestic investment (0.98); Commercial Durables: Yearly change in private inventories (0.48); Housing Starts: **New housing units per capita** (0.34); Infrastructure Investment: Per capita investment in private equipment (0.23); Per capita in private construction (0.97); *Per capita in public equipment (−0.80)*; Per capita in public construction (0.84)

Consumption	
PC1: 0.77	Cost of Living: Average consumer price index (0.93); Discretionary Spending: Personal discretionary spending (0.97); Goods and Services: Personal durable goods consumption (0.59); Personal non-durable goods consumption (0.99); Personal services consumption (0.99); Sustainable Consumption: Per capita spending on agricultural marketing (0.69)

Employment	
PC1: 0.26	Employment: ***Percent employed*** *(−0.69);* Unemployment**: Percent unemployed** (0.75); Employment Inequality: **Ogive Index** (0.62)
PC2: 0.23	Employment: **Manufacturing employees per capita** (0.58); Manufacturing employees per capita (0.60); *Percent self-employed (−0.62)*

Finance	
PC1: 0.78	Governance: *Total domestic revenues per capita (−0.98)*; *Gross public debt per capita (−0.93)*; Loans: Commercial loans per capita (0.95); Individual loans per capita (0.88); Real estate loans per capita (0.98); Savings: Personal savings per capita (0.99); ***Finance employees per capita*** *(−0.15)*

Innovation	
PC1: 0.51	Investment: Research investment per capita (0.72); Patents: *Patents per capita (−0.72)*

Production	
PC1: 0.54	Exports: Exports as percent GDP (0.25); Services: Volunteer participation per capita (0.95); Market Goods and Services: Yearly change in GDP (0.59); Sustainable Production: *Expenditures on renewables per capita (−0.92)*

Re-Distribution	
PC1: 0.32	Income Equality: **GINI Index** (0.62); Public Support: **Percent population using food stamps** (0.81); **Percent population receiving cash assistance** (0.75)
PC2: 0.23	Public Support: *Childcare funding per capita (−0.54)*; **Percent population on social security** (0.81)

**Table A4.** Metrics contributing most strongly to each principal component (PC) for Ecosystem Services, with metrics available at census-tract scale in bold, standardized loadings with that PC in parentheses, and italics indicating negative correlation with PC. Indicator categories for each metric from the original HWBI (Smith et al. 2012) are underlined

Service PC Variance Explained	Metrics

Air Quality	
PC1: 0.62	Usable Air: Percent canopy cover (air pollutant removal) (0.95); **Percent canopy cover in developed space** (**urban heat reduction**) (0.95); Percent good Air Quality Index (0.23)

Food/Fiber Provisioning	
PC1: 0.25	Food/Fiber: Total factor productivity (0.91); Food production index (0.74); Raw Material: *Metric tons of clay, salt, stone mining (−0.84)*
PC2: 0.19	Food/Fiber: **Crop production index** (0.88); **Percent prime farmland** (0.81)
PC3: 0.18	Food/Fiber: Percent farmland (0.70); Land area suited for forestry (0.77); **Percent forest cover** (0.64)
PC4: 0.12	Food/Fiber: Fish and shellfish landings (0.96)

Greenspace	
PC1: 0.56	Natural Areas: **Percent natural area** (0.93); Annual visitors to national parks (0.21); Recreation: **Species richness** (0.90); **Species richness** (0.95); ***Water area per capita*** *(−0.38)*

Water Quality	
PC1: 0.36	Usable Water: **Percent waterbodies assessed as good** (0.70); **Relative erosion potential** (0.76); Percent good beach days (0.44)

Water Quantity	
PC1: 0.38	Available Water: ***Percent area not in flood zone*** *(−0.80);* **Available water storage** (0.79); **Percent area not drought vulnerable** (0.71)
PC2: 0.22	Available Water: *Number of flood events (−0.61);* Percent days with no drought risk (0.81)

**Table A5.** Metrics contributing most strongly to each principal component (PC) for Social Services, with metrics available at census-tract scale in bold, standardized loadings with that PC in parentheses, and italics indicating negative correlation with PC. Indicator categories for each metric from the original HWBI (Smith et al. 2012) are underlined

Service PC Variance Explained	Metrics

Activism	
PC1: 0.50	Participation: Individual donations to political organizations (0.71); **Religious/civic/similar service employees per capita** (0.71)

Communication	
PC1: 0.58	Accessibility: Percent population with cell phone subscription (0.94); **Percent households with telephone** (0.83); Infrastructure: Percent households with broadband (0.96); Public Services: *Number of promotion projects (−0.87)*; Federal spending per capita on public broadcasting (0.62); *Spending per capita on public broadcasting (−0.83)*; Quality: *Voice and Accountability Index (−0.90)*
PC2: 0.14	Providers: Information employees per capita (0.96); **Information employees per capita** (0.13);

Community Initiatives	
PC1: 0.30	Investment: Federal spending per capita on arts and museums (0.86); Arts and culture employees per capita (0.87)
PC2: 0.29	Investment: **Arts and culture employees per capita** (0.85); *Spending per capita on film industry (−0.84)*
PC3: 0.20	Providers: Non-profits per capita (0.99);

Educational Services	
PC1: 0.28	Investment: Education expenditures per capita (0.90); Federal education spending per capita (0.88); Per pupil spending (0.93); Providers: Education employees per capita (0.58); Accessibility: Percent students receiving financial aid (0.53)
PC2: 0.16	Providers: **Education, Health, Social employees per capita** (0.79); **Education, training, and library employees per capita** (0.73); Accessibility: Percent schools with alternate education (0.60)
PC3: 0.13	Providers: Teachers per capita (0.71); Confidence: *Student to teacher ratio (−0.88)*
PC3: 0.12	Confidence: Cumulative Promotion Index (0.88); *Accessibility**: Public schools per capita (−0.73)*

Emergency Preparedness	
PC1: 0.37	Pre-disaster Planning: *Flood insurance policies per capita (−0.84)*; Insurance employees per capita (0.94); **Insurance and finance employees per capita** (0.11); Federal non-disaster emergency management spending per capita (0.95); Responders: **Protective service employees per capita** (0.14); Post-disaster Response: *Public Health Emergency Preparedness funding per capita (−0.91)*
PC2: 0.23	Responders: Emergency employees per capita (0.88); Public safety budget per capita (0.76); Post-disaster Response: Dollars per flood claim (0.48); *Federal spending on disaster response per capita (−0.71)*;

Family Services	
PC1: 0.29	Accessibility: Days to child services (0.82); Percent of homeless with shelter (0.70); Effectiveness: *Percent of adoptions taking less than one year (−0.57);* Providers: Family services employees per child (0.87); **Education, Health, and Social services employees per capita** (0.43); **Community and social services employees per capita** (0.11)
PC2: 0.28	Effectiveness: Rate of preventative services (0.65); Percent without a maltreatment recurrence (0.90); Investment: Federal spending on child welfare per capita (0.72); Spending on public welfare per capita (0.67); Providers: Family services employees per capita (0.86)

Healthcare Services	
PC1: 0.35	Investment: Federal spending on health and human services per capita (0.91); Health expenditures per capita (0.92); Providers: Health employees per capita (0.95); Quality: Percent treated properly for heart attack (0.62); Percent treated properly for heart failure (0.40); Percent treated properly for pneumonia (0.60); Percent treated properly for surgical infection (0.40); Accessibility: Medicare enrollees per capita (0.96);
PC2: 0.13	Accessibility: Health facilities per capita (0.86); **Percent population with Medicare** (0.77);
PC3: 0.13	Providers: ***Health, Education, or Social employees per capita*** *(−0.49);* ***Health practitioners or support per capita*** *(−0.57);* Accessibility: **Percent insured** (0.78); Health Professional Shortage Areas Score (0.37);

Justice	
PC1: 0.29	Environmental Justice: Federal environmental grants per capita (0.72); Investment: Justice and public safety spending per capita (0.91); Federal environmental financial aid per capita (0.90); Environmental spending per capita (0.89); Quality: Percent convictions in criminal cases (0.70); *Rule of Law Index (−0.69)*;
PC2: 0.17	Accessibility: Clearance rate of appellate cases (0.60); Investment: Federal justice spending per capita (0.89); Providers: *Justice employees per capita (−0.55)*;
PC3: 0.10	Accessibility: Clearance rate of trial cases (0.74); Environmental Justice: ***Population within 1000 m of toxic release facilities*** *(−0.46)*; Providers: **Government employees per capita** (0.26); **Legal service employees per capita** (0.76); Quality: *Percent convictions in criminal cases (−0.16)*;

Labor	
PC1: 0.64	Confidence: *Federal labor financial aid per capita (−0.51)*; Effectiveness: **Rate of injuries to workers** (0.80); Rate of injuries to workers (0.93); Rights: *Percent employees filing Equal Employment Opportunity charges (−0.88)*

Public Works	
PC1: 0.31	Accessibility: ***Percent using public transportation*** *(−0.38)*; Investment: Federal transportation financial aid per capita (0.65); Bus and maritime spending per capita (0.93); National park spending per capita (0.58); Federal highway financial aid per capita (0.66); Highway spending per capita (0.90); Public utility spending per capita (0.92); Providers: *Utility and transportation employees per capita (−0.88)*; Quantity: *Percent of time not under outage (−0.84)*; Quality: *Airport passengers per year (−0.87)*
PC2: 0.13	Accessibility: *Water quality violations per facility (−0.41)*; Investment: Federal environmental financial aid per capita (0.66); *Federal telecommunications financial aid per capita (−0.51)*; Quantity: Percent of landfill waste diverted (0.75); Fraction of roads in good condition (0.25)
PC3: 0.09	Providers: ***Utility and transportation employees per capita*** *(−0.34);* Quantity: Percent of bridges with no structural deficiencies (0.89); *Recycling facilities per capita (−0.90)*; Quality: Percent of bridges not functionally obsolete (0.23); Traffic per km of road (0.16)

**Table A6.** Estimated model parameters from full model averaging for well-being Domain metrics, reduced to Principal Components (columns; Appendix A Table A2) as a function of Services metrics, reduced to Principal Components (rows; Tables A3–A5). Empty cells indicate variable was not included in any of the top models by model selection. Shading indicates percent contribution of each variable to fixed R, estimated by Shapley scores, as described in key

	Key to Shading	Connect to Nat. PC1 (*Biophilia*)	Cultural Fulfill. PC1 (*Lack of arts attend.*)	Education PC1 (*Basic skills*)	Education PC2 (*Attainment*)	Education PC3 (*Social development*)	Health PC1 (*Life expectancy*)	Health PC2 (*Disease rates*)	Health PC3 (*Mortality rates*)	Leisure Time PC1 (*Work hours*)	Leisure Time PC2 (*Vacation*)	Living Standards PC1 (*Income and wealth*)	Living Standards PC2 (*No fear of job loss*)	Safety/Security PC1 (*Crime rate*)	Safety/Security PC2 (*Accidental Death*)	Social Cohesion PC1 (*Social engagement*)	Social Cohesion PC2 (*Trust in government*)	Social Cohesion PC3 (*Voter turnout*)
	1–10% of R^2^																	
	10–20% of R^2^																	
	20–30% of R^2^																	
	30–40% of R^2^																	
	>40% of R^2^																	
	>60% of R^2^																	

	**Numb. of Models**	407	11	2	4	1	8	12	9	2	31	1	2	7	447	126	1	2
	**R Fixed**	0.01	0.04	0.48	0.49	0.39	0.33	0.08	0.24	0.48	0.54	0.49	0.12	0.11	0.54	0.11	0.41	0.54
	**R Total**	0.1	0.4	0.99	1.00	1.00	0.99	0.15	0.30	1.00	0.98	1.00	1.00	0.18	0.97	0.23	0.86	1.00

	**% Contribution of**																	
	**Economic Serv.**	40.7	19.1	1.2	45.7	63.7	51.2	10.8	0.0	2.1	66.2	45.9	2.3	26.2	98	28.6	4.6	42.4
	**Ecosystem Serv.**	38.1	40.1	6.2	6.7	2.8	0.0	30.8	21.6	5.5	0.9	8.0	7.2	29.3	0.5	32.0	2.3	3.7
	**Social Serv.**	21.2	40.9	92.6	47.6	33.5	48.8	58.4	78.4	92.4	32.9	46.1	90.6	44.5	1.5	39.3	93.1	53.9

	**Intercept**	−0.09**	0.11 ^NS^	0.13 ^NS^	0.15 ^NS^	0.09 ^NS^	0.02 ^NS^	−0.02 ^NS^	−0.02 ^NS^	−0.03 ^NS^	0.01 ^NS^	0.00 ^NS^	0.01 ^NS^	0.00 ^NS^	0.00 ^NS^	−0.13**	0.05 ^NS^	0.05 ^NS^

**Economic Services**	**Capit. Invest. PC1**	0.00 ^NS^													0.00 ^NS^			
	**Consumpt. PC1**										**0.46** ***							
	**Employ. PC1**	0.02 *	0.06 ***					0.04 ***	0.00 ^NS^					0.05 ***	−0.01 ***	0.10 ***	−0.03 ***	
	**Employment PC2**	0.00 ^NS^			0.00 ^NS^				0.09 ***	0.05 ***	−0.01 ***		−0.01 ***	−0.07 ***	0.00 ^NS^			
	**Finance PC1**				5.71 ***	1.36 ***	−1.14 ***				−0.40 ***	6.77 ***			−0.02 ^NS^			−2.62 ***
	**Innovation PC1**	0.00 ^NS^	−0.05 ***		0.15 ***	0.03 ***		−0.04 *	0.00 ^NS^			0.13 ***		−0.05 ***		−0.03*		
	**Production PC1**														**-0.43** ***			
	**Re-distrib. PC1**	−0.04 ***	0.02 ^@^					−0.06 ***	−0.18 ***				0.003 ***	0.12 ***	0.01 ***	0.11 ***	0.08 ***	−0.09 ***
	**Re-distrib. PC2**	0.04 ***	0.03 ***	−0.06 ***			−0.02 ***	0.03 **	0.00 ^NS^						0.00 ^NS^	−0.06 ***	−0.03 ***	

**Ecosystem Services**	**Air Quality PC1**	0.00 ^NS^	0.01 ^NS^	−0.05 ***			0.00 ^NS^			0.00 ^NS^	0.01 ***	−0.02 ***	0.003 ***			0.03 ***		
	**Food/Fiber PC1**	0.00 ^NS^						0.16 ***	0.14 ***					−0.13 ***	−0.03 ***			
	**Food/Fiber PC2**	0.00 ^NS^	−0.03 ***	0.10 ***	−0.03 ***	−0.03 ***		−0.01 ^NS^			0.00 ^NS^	−0.09 ***	−0.01 ***				0.12 ***	0.05 ***
	**Food/Fiber PC3**	0.00 ^NS^		0.11 ***	−0.18 ***	−0.05 ***			0.05 ***	−0.09 ***	0.01 ***	−0.17 ***	−0.01 ***	−0.04 ***	0.00 ^NS^			0.09 ***
	**Food/Fiber PC4**	0.06 ***	0.06 ***					0.06 ***	0.00 ^NS^				0.004 ***		0.00 ^NS^	0.01^NS^	0.02 **	
	**Greenspace PC1**	0.00 ^NS^	0.05 ***								0.00 ^NS^					−0.12 ***		
	**Water Qual. PC1**	0.00 ^NS^	0.00 ^NS^				−0.02 ***	0.01^NS^	0.03 **	−0.05 ***	0.00 ^NS^		0.00 ^NS^	−0.01^NS^	−0.004 **	−0.01^NS^	−0.04 ***	0.00 ^NS^
	**Water Quan. PC1**	0.00 ^NS^	0.01 ^NS^		0.01^NS^		0.00 ^NS^	0.02^NS^	0.01 ^NS^	−0.02 **	0.00 ^NS^	0.07 ***	0.003 **		−0.01 ***	0.04**	−0.07 ***	−0.02 ***
	**Water Quan. PC2**	0.00 ^NS^	0.03 **		0.04 ***	0.01 ***		0.09 ***			0.01 ***	0.08 ***	0.004 ***	−0.03**	−0.01 *	−0.01^NS^	−0.04 ***	−0.02 ***

**Social Services**	**Activism PC1**	0.00 ^NS^						0.07 ***			0.01^NS^		0.01 ***	0.06**	−0.01 *	0.11 ***		
	**Communicat. PC1**									−0.3 ***					0.02 **	0.06 *		
	**Communicat. PC2**	0.01 ^NS^		0.53 ***				0.00^NS^					−0.05 ***		0.00 ^NS^	0.05 ^NS^		
	**Comm. Init. PC1**	−0.03 **	−0.03 ***						−0.20 ***	0.18 ***				0.12 ***	0.00 ^NS^	0.01 ^NS^	0.08 ***	
	**Comm. Init. PC2**	0.01 ^NS^	0.00 ^NS^		−0.12 ***	−0.04 ***	0.02 **	0.09 ***			0.02 ***	−0.12 ***	0.00 ^NS^		0.00 ^NS^	−0.03 ^NS^		0.07 ***
	**Comm. Init. PC3**	0.00 ^NS^		0.12 ***		−0.02 ***	0.00 ^NS^	−0.04**	−0.08 ***		−0.01**		−0.003 ***	0.06 ***	0.00 ^NS^	0.07 ***		
	**Education PC1**	0.00 ^NS^							−0.11 ***				−0.01 ***		0.00 ^NS^		−0.10 ***	
	**Education PC2**	−0.03 ***	−0.02 **	−0.06 ***	0.35 ***	0.08 ***		−0.08 ***	−0.13 ***	0.34 ***		0.32 ***		0.02 ^@^	0.00 ^NS^	0.00 ^NS^	0.11 ***	−0.20 ***
	**Education PC3**	0.00 ^NS^					0.00 ^NS^	−0.02 ^NS^	−0.04 ***					−0.01 ^NS^	0.00 ^NS^	−0.02 ^NS^		
	**Education PC4**	0.00 ^NS^	−0.02**				0.01 ***	−0.03 *			0.01 **	−0.06 ***		−0.05 ***	0.00 ^NS^	−0.06 ***	0.05 ***	
	**Emerg. Prep. PC1**				−0.57 ***	−0.10 ***	0.20 ***				0.13 ***	−0.60 ***		−0.14 ***	−0.02 ^NS^			0.20 ***
	**Emerg. Prep. PC2**	0.00 ^NS^		−1.90 ***											−0.01 ^NS^	0.00 ^NS^		
	**Family Serv. PC1**	0.00 ^NS^		−0.31 ***	0.88 ***	0.19 ***						0.36 ***			0.00 ^NS^	−0.01 ^NS^		−0.26 ***
	**Family Serv. PC2**	0.00 ^NS^								0.58 ***	−0.13 ***				0.00 ^NS^	−0.03 ^NS^		
	**Healthcare PC1**								−0.14 ***					0.09 ***	0.00 ^NS^			
	**Healthcare PC2**	0.00 ^NS^	0.06 ***	−0.08 ***			0.01 ***	0.00 ^NS^	0.10 ***	−0.01**	0.00 ^NS^		0.004 ***	−0.12 ***	0.01 ***	−0.13 ***	−0.11 ***	
	**Healthcare PC3**	0.00 ^NS^	0.04 ***	0.02 ^NS^	−0.11 ***	−0.03 ***				−0.09 ***	0.00 ^NS^	−0.04 ***			0.00 ^NS^		−0.10 ***	0.05 ***
	**Justice PC1**						**0.48** ***			−3.82 ***	0.32 ***		**0.2** ***		−0.06 ***			
	**Justice PC2**	0.00 ^NS^	−0.01 ^NS^												0.00 ^NS^	0.01 ^NS^	0.92 ***	
	**Justice PC3**	0.00 ^NS^	−0.05 ***				−0.02 ***	−0.08 ***							0.00 ^NS^	−0.04 ***	0.10 ***	
	**Labor PC1**	0.00 ^NS^	−0.02**				0.05 ***		0 11 ***					0.01 ^NS^	0.00 ^NS^	0.04 ^NS^		
	**Pub. Works PC1**			−0.98 ***	0.70 ***	0.31 ***	−0.14 ***					1.00 ***	0.01**		−0.01 ^NS^	0.00 ^NS^		−0.55 ***
	**Pub. Works PC2**	0.00 ^NS^	0.01 ^NS^	0.05 ^NS^		−0.04 ***		−0.05 *		0.16 ***					0.00 ^NS^	0.00 ^NS^		
	**Pub. Works PC3**	0.00 ^NS^	0.00 ^NS^							0.12 ***					0.00 ^NS^			

Variable significance indicated as

*** *p* < 0.001

** 0.001 < *p* < 0.01

* 0.01 < *p* < 0.05

@ 0.05 < *p* < 0.1

NS, not significant. The primary (but not exclusive) metric(s) loading onto each domain PC is given in italics in each column heading.
